# Plant Nanomaterials and Inspiration from Nature: Water Interactions and Hierarchically Structured Hydrogels

**DOI:** 10.1002/adma.202001085

**Published:** 2020-06-14

**Authors:** Rubina Ajdary, Blaise L. Tardy, Bruno D. Mattos, Long Bai, Orlando J. Rojas

**Affiliations:** ^1^ Department of Bioproducts and Biosystems School of Chemical Engineering Aalto University P.O. Box 16300, Aalto Espoo FIN‐00076 Finland; ^2^ Departments of Chemical & Biological Engineering Chemistry and, Wood Science The University of British Columbia 2360 East Mall Vancouver BC V6T 1Z3 Canada

**Keywords:** biocolloids, biohydrogels, hydrogels, nanocelluloses, plants, porous materials, structuring, water interactions, wood

## Abstract

Recent developments in the area of plant‐based hydrogels are introduced, especially those derived from wood as a widely available, multiscale, and hierarchical source of nanomaterials, as well as other cell wall elements. With water being fundamental in a hydrogel, water interactions, hydration, and swelling, all critically important in designing, processing, and achieving the desired properties of sustainable and functional hydrogels, are highlighted. A plant, by itself, is a form of a hydrogel, at least at given states of development, and for this reason phenomena such as fluid transport, diffusion, capillarity, and ionic effects are examined. These aspects are highly relevant not only to plants, especially lignified tissues, but also to the porous structures produced after removal of water (foams, sponges, cryogels, xerogels, and aerogels). Thus, a useful source of critical and comprehensive information is provided regarding the synthesis of hydrogels from plant materials (and especially wood nanostructures), and about the role of water, not only for processing but for developing hydrogel properties and uses.

## Graphical abstract

Inspired from nature, wood‐based and man‐made hydrogels are produced taking advantage of the properties and structure of elements present in the cell walls of plants, including (nano)celluloses. They endow new materials with features that include directionality, hierarchy, responsiveness, and function, all of which are associated to the composition and morphology of the building blocks.

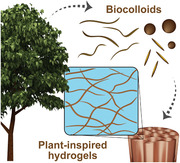

## 1 Introduction

Hydrogels are versatile materials that find use in food, pharma, medicine, engineering, and agriculture. The design of multifunctional hydrogels is emerging as an answer to meet present and future challenges associated to environmental remediation, sensing, drug delivery, biomedicine and packaging, among many others. Considering the adoption of the circular bioeconomy, bio‐based materials are becoming increasingly important. In these efforts, hierarchical structures found in nature can serve not only as a source of inspiration but to endow hydrogels with unique features; these subjects have become the focus of recent attention by the scientific communities. Advances offering the “green” promise are intimately linked to water, which is essential in our determination to reduce environmental impacts by favoring uses of renewable sources. Therefore, the topic of “hydrogels” is becoming extremely relevant as new plant‐based polymers find expanded utilization. Specifically, wood‐derived nanomaterials are asserted for their merits in the synthesis of hydrogels (biohydrogels). Recent breakthroughs involve natural polymers such as polysaccharides (cellulose, starch, pectin, alginate, chitosan), proteins (gelatin, silk fibroin, egg albumin, casein), and lipids (vegetal oil, fatty acids), among others. Such biopolymers display many valuable properties that strongly depend on the interactions with water, which plays a key role during their extraction and, importantly, in any effort associated with their use or in their assembly into new structures. The subject of this review is this latter aspect, in the context of plant‐based hydrogels. We start describing the multiscaled nature of wood‐derived (nano)materials and their close relation with water. Following, we introduce the main plant‐centered sources of hydrogels, principally wood, and the significance of solubility, capillarity, and diffusion phenomena. They are all relevant to bio‐inspired hydrogels and their processing. For this, we briefly discuss the use of hydrogels in spinning and 3D printing, regeneration and coagulation as well as biofabrication. The review presents the means to re‐engineer biologically inspired hydrogel architectures that feature anisotropy (nematic and chiral nematic). In this framework, the role of ions in aqueous media cannot be ignored. Other themes include (bio)mineralization using ion‐infused and ion‐crosslinked nanocellulosic hydrogels. Our contribution continues describing multicomponent and nature‐inspired hydrogel systems. The latter are presented aiming to connect the properties of the resultant materials with those of the natural precursor, in its natural state. Here we yet again, consider water and its interactions. We end by briefly describing the prospects of other plant‐based hydrogel precursors, different than cellulose or its nanostructures.

## 2 Definitions

Hydrogels are 3D macromolecular networks that display high water binding and retention. The topology of a hydrogel strongly depends on its building blocks, their size and the history of formation, where thermodynamic effects occurring prior and during assembly may lead to substantially different architectures. For instance, this is illustrated by the dynamics of sol–gel transitions^[^ ^1^ ^]^ and thermodynamic equilibrium,^[^ ^2^ ^]^ which determine the main mechanical attributes and superstructures that develop in the given hydrogel. Gel formation occurs through weak and strong interactions, tethering the properties of the constructs. Weak interactions can be summed to form strong hydrogels such as those including double and triple helices, while increasing reversibility of links, mostly from temporal interactions involving hydrogen bonding, ionic association, and binding ((block) copolymer, micelles and others), result in weaker yet more dynamic hydrogels. Chemical and covalent gelation is achieved by various chemical processes including condensation, vulcanization, and addition polymerization.^[^ ^3^ ^]^ In relation to these phenomena, hydrogels can be characterized by their rheological behavior, which also depends on the process used in their synthesis. The hydrogel rheology, for instance, affects its strength, which to some extent defines its use.^[^ ^4^ ^]^ As will be discussed later in this review, cellulose nanomaterials (nanocelluloses) display a behavior that can be advantageous in the generation of hydrogels, depending on their concentration in aqueous media, going from liquid suspensions, at low mass loadings, to strong solid‐like hydrogels over a critical concentration.^[^ ^5^, ^6^ ^]^

**Figure 1:**
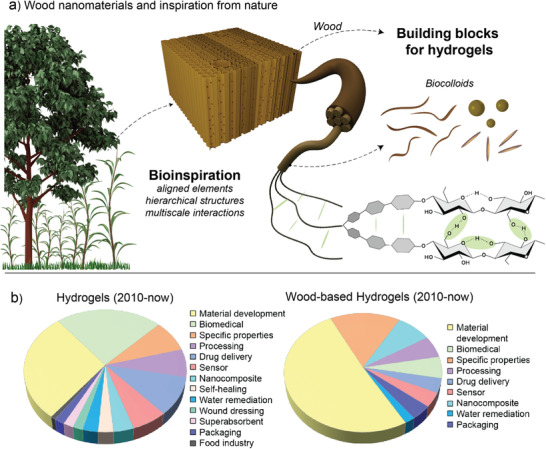
a) Inspired from nature, man‐made hydrogels are produced from elements extracted from the cell walls of plants, here represented by vascular plants (trees, grasses). Processing in water of plant‐based biocolloids endow hydrogels with special features that include “nature’s memory” as far as directionality, hierarchy, responsiveness, and function, all of which relate to the composition and morphology of the building blocks. The latter, termed as “biocolloids” can include polymeric assemblies derived from cellulose (see molecular structure) as well as noncellulosic components such as hemicelluloses, lignin and others. b) Subject areas of papers published since 2010 in the field of “hydrogels” in general (left), and hydrogels derived from wood‐based components (right) (Scopus search with a combination of related keywords, accessed December 2019).

As we start this review, it is important to place the subject of “hydrogels” in the context of their applications, which are wide ranged but, inherently, share some common aspects. They include, for instance, the fact that hydrogels form self‐standing structures, are water‐swollen, and easily form 3D networks. Such features are very relevant to life sciences but, have been more recently recognized for their potential impact in (cellulosic) fiber technologies. Water is strongly associated with plants and is the main medium used for processing of plant components such as fibers at the macroscale as well as fibrils and crystals at the colloidal and nanometric scales. Indeed, fiber handling and structuring in nonwovens, such as paper, entails suspending the materials in water, their fibrillation, refining, screening and then filtration, pressing and eventually drying. Water and water interactions are essential in each of these steps. As it is clear now, plants, especially wood, provide the building blocks used to synthesize hydrogels. On the other hand, it is remarkable that plants can also serve as source of inspiration for the design of nanostructured materials, displaying features such as anisotropy and compartmentalization as well as unique functions (transport and others) (**Figure** **1**a). It is not surprising that many recent reports relate to hydrogels produced from nanomaterials derived from wood, especially for use in biomedical materials where the biological compatibility of cellulose and its cell adhesion properties have been noted as prime advantages. Additional aspects emerge from their inherent high mechanical strength, reaching that of “wet” architectures found in nature. Hence, expectedly, wood‐derived hydrogels show promise in replicating the complex interactions between water and typical biological systems, such as those found in plants as well as across the animal kingdom, for instance, in cartilages and bones. The applications of hydrogels in general (polymeric, synthetic, colloidal, etc.) overlap with those prepared specifically from wood‐based materials (see Figure 1b for a comparison of main uses considered in the scientific literature since 2010). The latter ones resemble some of the inherent properties of the source materials, as noted from the point of view of anisotropy (directionality), hierarchy, responsiveness, and function. For these very reasons, we discuss next the nature of water interactions and their relevance to wood (nano)technologies to then expand into the biological sources of hydrogels.

## 3 Multiscaled Water Interactions in Wood (Nano)Technologies—A General Perspective

Here, we briefly summarize key developments in wood technologies, from the macroscale to the nanoscale, and how the ubiquitous relationship between wood and water has been central to the progress in this field. Indeed, wood has been a key structural material from preagricultural times, given its availability, light‐weight and strength, among others. Early on, efforts were directed to understanding the role of water, for example, to bend wet wood into predesigned shapes. At present, the effects of wood wetting, as far as the compression and flexural strength as well as the elastic moduli, are well described. In contrast, the involved molecular‐level phenomena are yet to be fully elucidated.

Fiber processing developed from the postagricultural era, with uses associated to the manufacture of textiles (e.g., flax fibers spinning into linen) and as support for communication, for example, the Chinese paper. In the latter case, water interactions with the fiber precursors have been key for efficient assembly. The first forms of papers were produced from aqueous slurries of recycled fibers (used textile rags made of hemp and flax) that consolidated into sheets by removal of water (upon dewatering, pressing and drying). Such papermaking processes gave birth to investigations on fiber behavior in aqueous suspensions (e.g., flocculation, sedimentation and entanglement), mostly to correlate processing and materials properties. More recent advances involve efficient mechanical (e.g., Asplund method) and chemical (e.g., Soda and Kraft) processes for making wood pulp from chips, resulting in purer and well dispersed fibers. The creation of efficient defibrillators made possible significant technological advances in the production of structural fiberboards (e.g., medium‐density fiberboard (MDF)). The optimized use of water was key in such efforts but it is only until recently that the connection with hydrogels, especially with the advent of cellulose nanomaterials, has become apparent.

Advances in fiber processing have incorporated highly intense deconstruction of wood structures into the nanoscale (10^−9^ m). Nanocelluloses, a term used here to describe nanocrystals and nanofibrils, have become quite prominent. The former type, cellulose nanocrystals (CNCs), were first observed in the 1950s during controlled acid‐catalyzed degradation of cellulose fibers.^[^ ^7^ ^]^ The cellulose nanofibrils (CNFs), were reported in the 1980s, when they were observed to form viscous gels after processing wood pulp in a milk homogenizer operated at high pressure.^[^ ^8^ ^]^ Water, as a reaction medium, induces fiber swelling and allows the diffusion of reactants (e.g., sulfuric acid) in the less‐ordered regions of the solid structure, yielding the needle‐like, highly crystalline CNCs.^[^ ^9^ ^]^ For CNFs, the presence of water is key for fiber swelling and to loosen the interfibril H‐bonding thus, allowing efficient defibrillation of wood pulp, while avoiding fibril damage. A striking fact, related to the subject of “hydrogels,” is the fact that only recently the surface of cellulose was described, most appropriately, as chains “like eel grass on the bottom of a pond”^[^ ^10^ ^]^ and later, associated to gel‐like, water‐swollen dangling tails (or molecular fibrils) on the surface of cellulose.^[^ ^11^ ^]^ These and other aspects related to the role of water in fiber processing can be found in some of our past reviews.^[^ ^12^, ^13^ ^]^

**Figure 2:**
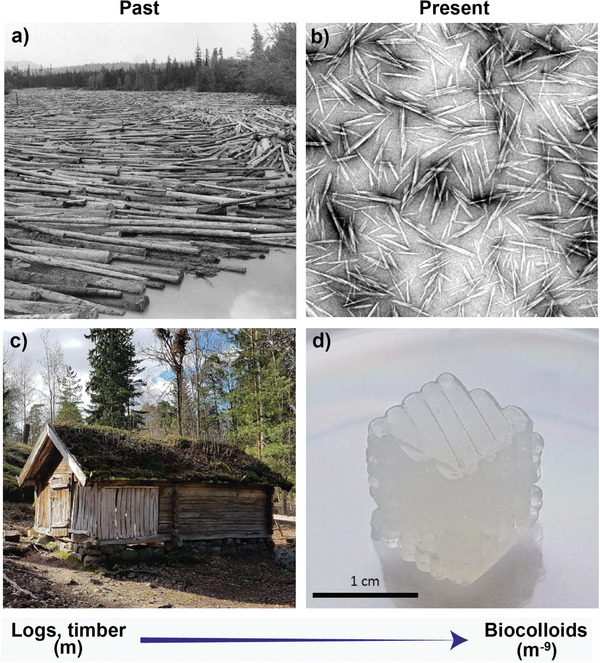
Illustration of the paradigm shift associated with the use of plant‐based materials, going from macroscaled, biologically structured lumber (a,c) to colloidally engineered nano‐ or biocolloids (b,d). a) Picture of logs in a river. Reproduced with permission from the Forestry History Society. b) TEM image of cellulose nanocrystals. Adapted with permission.^[^ ^16^ ^]^ Copyright 2008, The Royal Society of Chemistry. c) Photo of a traditional Finnish wooden cabinet. Reproduced with permission from Katariina Solin. d) 3D printed hydrogel from a cellulose nanocrystals (CNC) suspension (personal archive).

As the isolation of CNC and CNF was only possible in the presence of water, the applications intended for such nanomaterials should also consider the water that is associated at various scales. Similar to traditional papermaking, efforts are aimed at reducing the consumption of water for nanocellulose production. Moreover, the knowledge that exists in the area of macrofiber networks (paper) is being translated to the field of nanocelluloses.^[^ ^14^ ^]^ Interestingly, many concepts related to water interactions apply to both scales; however, with additional considerations arising from the high surface area of the nanocelluloses, scaling factors account for a high density of interfibril connections. This puts the associated physics in the realm of colloidal interactions, which demand the use of sophisticated measurement techniques^[^ ^15^ ^]^ to reveal the role of water, especially pertinent to the synthesis of hydrogels. These aspects are shown in **Figure** **2**, which schematically illustrates the correlation between macrostructures, as known from the past, and the emerging nanomaterials, in the context of hydrogels.

## 4 Hydrogel‐Like Structures Found in Nature

In this section we discuss hydrogel‐like structures that are found in multicellular organisms, such as plants, which at a given stage of development or in given conditions contain, form or are associated with hydrogels. For instance, vascular and nonvascular (e.g., algae) plants can be considered as hydrogels at some stage of their development. Such “living” hydrogels fulfill given functions that could be used to inspire the design of man‐made counterparts. In this review, we consider not only hydrogels made form components extracted from plants and also suggest the interesting possibility of those that are designed following the structures found in nature, for example, to achieve unique functions. The basic definition of a hydrogel relates it to an “interconnected network that swells in water but keeps its integrity.”^[^ ^17^ ^]^ This concept applies to a large number of organisms and structures found in nature. Animals, microorganisms and plants often display characteristics that fit this definition.^[^ ^18^ ^]^ In the animal kingdom, jellyfish (a general term that applies to species from the phylum *Cnidaria*) have been highlighted as living hydrogels with an invertebrate body composed of ≈95% of water.^[^ ^19^, ^20^ ^]^ Many bacteria can secrete polysaccharide‐based hydrogels in the form of biofilms.^[^ ^21^ ^]^ Typical examples are the *Azotobacter vinelandii* that secretes alginate hydrogels as part of its defense and survival mechanisms;^[^ ^22^ ^]^ likewise, as a result of its metabolisms, *Komagatiaeibacter* sp. secrete cellulose nanofibers (bacterial nanocellulose, BCN) forming a strong hydrogel pellicle.^[^ ^23^, ^24^ ^]^ Such hydrogels, originated from vegetal or animal sources, are of special interest due to their better acceptance compared with petrochemically derived systems. Moreover, the availability and facile manipulation of the primary building blocks (biocolloids and biopolymers) are major advantages. Despite their generic botanical dissimilarities, both terrestrial and aquatic plants possess a remarkable ability to support very large volumes of water, without losing tissue cohesion. Following such observations, we emphasize the similarity between plants and man‐made hydrogels, even if the relatively low porosity of the former ones^[^ ^25^ ^]^ make them to swell to a more limited extent (for example, ≈15% in their natural form,^[^ ^26^ ^]^ compared to typical man‐made cellulosic hydrogels, over 10^5^%).^[^ ^27^ ^]^ In the next section we focus on a particular case relevant to wood, in its natural form, which can be processed into a structure that becomes a hydrogel when in contact with water (wood hydrogel). We then shift our attention to hydrogels produced from the elements extracted from wood or plants (plant‐based hydrogels).

### 4.1 Wood Hydrogels

**Figure 3:**
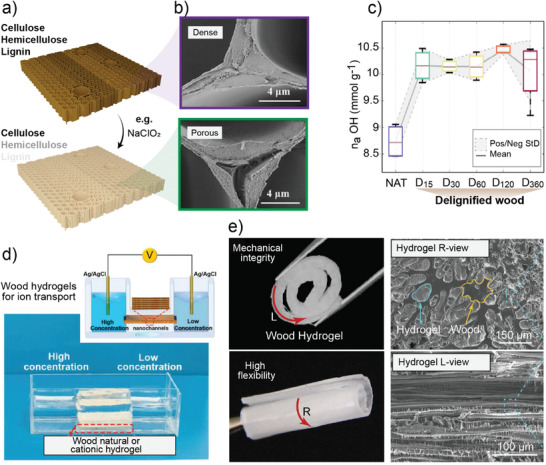
a) Lignin and hemicelluloses are selectively removed from solid wood by a variety of chemical routes (e.g., using NaClO_2_). This allows the preparation of structured scaffolds and hydrogels. b) Removal of lignin, especially from the middle lamellae, increases the porosity of the material, as shown in the SEM images of the middle lamellae and c) subsequently increases water binding sites. In (c) natural wood (NAT) was delignified using H_2_O_2_/acetic acid during given reaction time (15–360 min, D15 to D360). a–c) Adapted with permission.^[^ ^25^ ^]^ Copyright 2019, American Chemical Society. Wood hydrogels (modified or not) contain aligned pores that can be used as nanofluidic devices for selective ion transport. An example is shown in (d) that includes an ion‐regulation device that shows given current–voltage profiles, as determined by Ag/AgCl electrodes placed on the two sides of the wood hydrogels. In such applications, ions of interest include Cl^−^ and K^+^. Reproduced with permission.^[^ ^32^ ^]^ Copyright 2019, Wiley‐VCH. e) Highly flexible wood hydrogels rolled in the longitudinal (L) and radial (R) directions upon alignment of anisotropic fibril elements that interact strongly with elastic co‐components. Adapted with permission.^[^ ^30^ ^]^ Copyright 2019, Wiley‐VCH.

The high‐ordered assembly in wood and the intimate chemical interactions between its hydrophilic and hydrophobic components (cellulose, hemicelluloses, lignin and extractives), modulate the interactions with water. Recently, strategies to achieve controlled delignification have been optimized to prepare wood‐like constructs (termed in some reports in the literature as “nanowood”) (**Figure** **3**a).^[^ ^25^, ^28^, ^29^, ^30^, ^31^, ^32^, ^33^, ^34^, ^35^ ^]^ Such efforts exploit the fact that the region between wood fibers (the middle lamellae) is highly concentrated in lignin while most of this biomolecule is dispersed in the cell wall, especially in the secondary layer (S2), acting as a glue for the fibrillar cellulose framework.^[^ ^36^ ^]^ Most of the delignification processes utilize chemical reagents (e.g., NaCl_2_, NaOH, Na_2_SO_3_) to remove up to 95% and 75% of the initial mass of lignin and hemicellulose present in wood, while maximizing the retention of cellulose.^[^ ^29^, ^33^, ^37^ ^]^ A well‐controlled removal of lignin from wood’s structure opens periodic vacancies across the whole material, therefore increasing its porosity (Figure 3b).^[^ ^25^ ^]^ In addition, a significant number of water‐binding sites become available for diverse interactions (Figure 3c).^[^ ^25^ ^]^ The integrity of the final cellulose scaffold can be maintained, at least to some extent,^[^ ^30^ ^]^ by taking advantage of the multiple and strong interfibrillar interactions that exist in the original, nature‐given structure. Such scaffolds swell with water following a directional hygroexpansion, as also observed in wood (ratio of longitudinal‐to‐cross‐sectional swelling >1000). During such hygroexpansion (wood or nanowood), the tangential and radial directions undergo major dimensional changes but relatively minor changes occur in the longitudinal one. The associated swelling/shrinkage behavior of the material allows the preparation of wood hydrogels with highly directional structural response to water. This differs drastically from typical polymeric structures—cellulosic or not—that are isotropic and swelling is nondirectional,^[^ ^27^ ^]^ unless the material is engineered for such purpose.^[^ ^38^ ^]^

The alignment of nano–microscale channels parallel to each other is a structural feature of wood hydrogels,^[^ ^30^ ^]^ which can be mimicked in typical isotropic systems only by external efforts (such as ice templating).^[^ ^39^, ^40^ ^]^ The channels within the microstructure of the hydrogel have limited interconnections (e.g., through pits in coniferous species), which can in fact be used to regulate fluid exchange in the radial direction.^[^ ^31^ ^]^ However, fluid transport within wood hydrogels mostly follows the longitudinal direction (parallel to the fibers’ principal axis), through the relatively large wood capillaries (lumens). This has been harnessed in nanofluidic devices used for highly efficient and selective ion transport (Figure 3d).^[^ ^32^ ^]^ Additionally, delignified wood density is increased by compression in the radial direction, thus creating nanochannels that exhibit improved ionic conductance (90 times higher) compared to the bulk solution (1.28 × 10^−3^ vs 1.4 × 10^−5^ S cm^−1^ for 1 × 10^−3^ m KCl solution).^[^ ^32^ ^]^ Although this concept has been tested only for few wood species, many opportunities can be expected by exploiting the unique features found in the types of wood that are available, each with a distinctive anatomy, not only in terms of morphology and dimensions but cell types and functions, which include tracheid elements (gymnosperms), fibers and vessels (angiosperms) and parenchymal cells.^[^ ^41^ ^]^ Hence, nature provides a readymade toolbox for the preparation of hydrogels with tailored transport (fluid, ion, heat, stress, etc.) and directional actuation.

From a mechanical perspective, wood hydrogels can outperform those that are man‐made. The strength of wood hydrogels (over 10^4^ kPa) is at least two orders of magnitude higher than that of a polyacrylamide hydrogel (below 10^2^ kPa).^[^ ^30^ ^]^ This arises from the multiscale and hierarchical structure of native wood that includes bundles of nanofibrils aligned in given directions with respect to the main axis of the plant. In addition to strength, remarkable flexibility and shape recovery properties are noted for wood hydrogels (Figure 3e).^[^ ^30^ ^]^ Such morphology‐centered features explains the emergence of applications related to cell culturing and tissue engineering,^[^ ^30^ ^]^ along with the fabrication of complex material designs.^[^ ^42^ ^]^

The evolutionary processes leading to the formation of hydrogels in nature highlight the importance of the elementary structure of the organism (animal, microorganism or vegetal) in holding and interacting with water while functioning or serving a given purpose. Structuring varies to a great extent among the organisms composed by or forming hydrogels; however, the hierarchical structures, particularly those found in vascular plants (e.g., wood), mostly comprising cellulose, raises a great interest for the formation of hydrogels. The impressive mechanical performance of wood’s primary building blocks as well as their facile processability has intensified their utilization for material development. Water is ubiquitous in cellulose biocolloids, in their natural state, for their isolation or for processing. Hence, before introducing plant‐based hydrogels, e.g., those formed from the components extracted from plants (for example the biocolloids shown in Figure 1a), we first discuss the subject of water interactions, which so far in this review has been indicated as essential for the synthesis, processing and application of hydrogels.

## 5 Water Interactions in Multiscaled Cellulosic Assemblies

**Figure 4:**
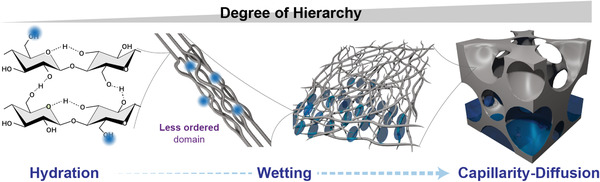
Some relevant physicochemical phenomena involving water and cellulose and its constructs (nanocelluloses, for example), including molecular interactions with hydroxyl groups within the supramolecular structure, wetting of their primary assemblies, and capillary intrusion followed by diffusion in high‐ordered materials built from cellulose.

Understanding the interactions of water with multiscale lignocellulosic elements, especially cellulose, is paramount in the development of next generation bio‐based materials. Water interacts with cellulose and its associated structures through physical and chemical mechanisms and across different length scales. They extend from (disordered or crystalline) macromolecular cellulose to bulk materials (e.g., wood, foams, and sponges). In this context, key phenomena deserving our attention include solubility, hydration, wetting, capillarity and diffusion (**Figure** **4**). They are all involved in processes associated with: a) the living hydrogels, as introduced in previous sections (for example, vascular plants, especially those with lignified tissue where capillary transport is paramount), b) wood hydrogels with their compartmentalized structures, and c) plant‐based hydrogels that are eventually subjected to drying, generating highly porous structures that offer efficient means storage, transport, or release of water (or other fluids).

### 5.1 Solubility

Cellulose insolubility has been discussed in terms of the multiple intra and interchain hydrogen bonds, its crystallinity, its amphiphilic character and hydrophobic interactions, all of which are the result of specific molecular conformations derived from the beta glycosidic bonds in the cellulose macromolecule.^[^ ^43^, ^44^, ^45^, ^46^, ^47^, ^48^ ^]^ The presence of well‐coordinated, short‐range H‐bonding across the polymer chains promote a very tight and ordered macromolecular arrangement, leading to a relatively high crystallinity. The amphiphilic character of cellulose also strongly impacts its interactions with water at a molecular scale. Within the cellulose macromolecule, there is a clear segregation of polar and nonpolar domains, in which the polymer chains are linked by in‐plane intermolecular hydrogen bonds while at the same time they stack perpendicularly, promoting hydrophobic interactions between C–H groups. Such features have been studied by molecular simulation with model cellulose crystals^[^ ^49^ ^]^ and by experimental observations on the chemical anisotropy of cellulose nanocrystals.^[^ ^9^ ^]^ Despite being far from totally understood, the work reported so far has built the foundation for the development of incredible materials built from the regeneration, assembly or gelation of solutions of cellulose and its derivatives as well as its colloidal suspensions.^[^ ^50^ ^]^

### 5.2 Hydration and Wetting

Water, in both the liquid and vapor states, forms a tightly bound layer on the surface of cellulose.^[^ ^51^, ^52^ ^]^ Such water, even if adsorbed as a monolayer, plays a key role during surface modification, e.g., by physical adsorption, covalent grafting or chemical vapor deposition.^[^ ^53^ ^]^ The states of water near the surfaces of cellulose can be thermodynamically considered as free, freezing, and nonfreezing. Briefly, free water is present in excess and possesses the same physicochemical properties of bulk water (with interactions taking place among neighboring molecules). Freezing water corresponds to those molecules confined within the ultrastructure (pores) of the cellulosic material in a way that it shifts the temperature for solid–liquid phase transition. Freezing water is within the range of a hydrogen bond (≈1.5–2.5 Å). Its peculiar behavior has been harnessed in the characterization of pores in nanocelluloses and related hierarchical structures.^[^ ^25^, ^52^, ^54^ ^]^ Lastly, nonfreezing water—also called bound water—corresponds to molecules adjacent to the surface, which cannot freeze due to dimensional and conformational restriction in water motion, arising from the strong interactions with cellulose. Bound water, especially, plays a key role in the hydration and wetting of nanocelluloses and therefore affects any material that comprises at least one phase in the aqueous state,^[^ ^55^, ^56^, ^57^ ^]^ as is the case of hydrogels.

**Figure 5:**
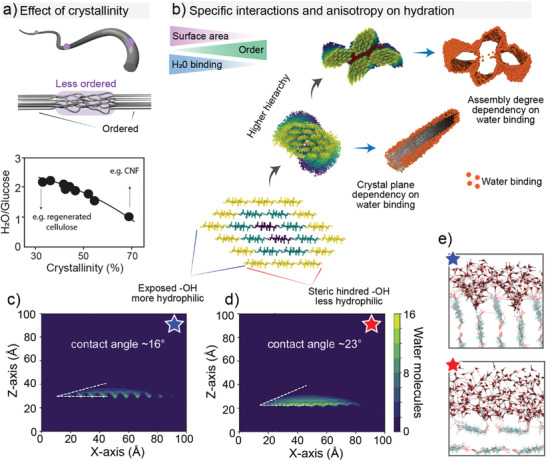
Interactions between water and cellulose at the supramolecular scale. a) Overall effect of the cellulose crystallinity on the amount of bound water, in which the supramolecular disorganization of the disordered regions of the cellulose crystal (graphically represented in the illustration) accounts for a higher water binding per glucose units. Adapted with permission.^[^ ^58^ ^]^ Copyright 1998, Elsevier. b) Schematic illustration of site‐specific water interactions as a function of the cellulose crystallographic planes and its higher order assemblies. The total water binding has a direct relationship with the surface area of the cellulose structure deriving from the amount of accessible hydroxyl groups, which decreases as a function of the hierarchical order in the assembly. Reproduced under the terms of the CC‐BY Creative Commons Attribution 4.0 International license (http://creativecommons.org/licenses/by/4.0/).^[^ ^59^ ^]^ Copyright 2019, The Authors, published by Springer Nature. c–e) Molecular dynamic simulations of the wetting of water nanodroplets on the crystallographic planes of cellulose. In (c) the cellulose crystallography plane exposed to interactions (110) displays more accessible water binding sites and rougher features, resulting in high water wetting. In (d), the 100 crystallographic plane of the cellulose lattice does not favor wetting due to steric hindrance, which leads to buried water binding sites. e) Details of the conformation of water molecules on and around the cellulose molecules on the surface of the wetting site. In (e) the top part refers to the (110) plane shown in (c), whereas the bottom part refers to the (100) plane displayed in (d). c–e) Reproduced with permission.^[^ ^49^ ^]^ Copyright 2020, Springer Nature.

There is a strong correlation between cellulose ultrastructure and hydration. Water binding depends on the cellulose crystallinity (**Figure** **5**a),^[^ ^58^ ^]^ crystallographic planes (Figure 5b–e)^[^ ^49^, ^59^ ^]^ and the degree of structuring in the multiscale constructs (Figure 5b).^[^ ^56^ ^]^ Variations on such features occur naturally (through biosynthesis) or upon processing (e.g., bottom‐up regeneration or top‐down nanocellulose isolation). It has been shown that a significant reduction in the number of bound water molecules (from >2 to <1 H_2_O by glucose unit) occurs as the crystallinity of cellulose increases from 30% to 70% (Figure 5a).^[^ ^58^ ^]^ More interestingly, the cellulose elementary fibril units (in botany referred to as microfibrils) possess an amphiphilic cross section with chemical anisotropy between the crystallographic planes (Figure 5b).^[^ ^49^, ^59^ ^]^ Recent molecular dynamic simulations indicate a cellulose I crystal plane that exposes hydroxyl groups (110), and is rough (at the molecular scale) (Figure 5c–e top panel). It has greater water binding and therefore better wetting compared to the 100 plane, which possesses “buried” hydroxyl groups (Figure 5d,e bottom panel). CNCs prepared from acid hydrolysis of plant fibers display such chemical anisotropy. Having such plane‐dependent wetting behavior has allowed the formation of very interesting multiphase systems and colloidal assemblies of CNCs at interfaces.^[^ ^9^, ^60^ ^]^

In plants, cellulose chains form twisted elementary fibrils (with a typical twist of 4° by nm) due to a spatial conformational response of the polymer, driven by energy minimization. The elementary fibrils, which are amphiphilic, further assemble into nanoscaled bundles with a transferred right‐hand twist along the bundle’s length (with twist rate of 0.9° by nm).^[^ ^59^ ^]^ The density of water binding in the bundles should be always lower than those observed at their primary units, mainly because of a simple relationship between specific surface area and accessible hydroxyl groups.^[^ ^56^, ^59^ ^]^ However, the mismatch in the twist rate between single fibrils and bundles creates periodic structural openings for water to interact with the inner parts of the bundles, thus creating a conformational disorder that favors swelling. Taking into consideration that water, as a solvent, does not interpenetrate the cellulose chains, the swelling of cellulosic materials is thought to arise from the diffusion of water molecules in‐between the primary building blocks. Such phenomenon is observed even in the simplest assembly—lowest hierarchy degree—of cellulose fibrils, namely elementary fibrils forming bundles; however, the swelling ratio tends to increase in line with the disorder of the construct (Figure 5b).^[^ ^61^ ^]^

**Figure 6:**
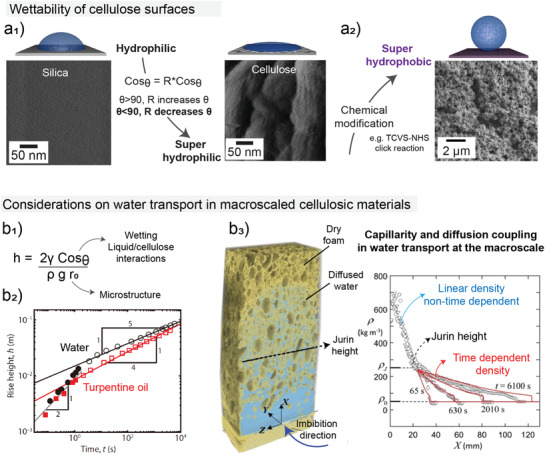
Water interactions with high order constructs based on cellulose as a precursor of an hydrogel or in porous supports produced after removal of water from cellulose‐based hydrogels. a_1_) Cellulose surfaces display a superhydrophilic character derived from the groups in their chemical structure and from the inherent roughness of the fibrils and their films. a_2_) Superhydrophilicity is converted to superhydrophobicity taking advantage of cellulose’s highly reactive and rough surface, which is easily modified. a) Adapted with permission.^[^ ^64^ ^]^ Copyright 2016, American Chemical Society. At a higher, scale capillary and diffusion drive the water transport in cellulose‐based, inherently porous materials. b_1_) The maximum rise length, the Jurin height, is strongly affected by the wetting of the cellulose by the wicking liquid as well as the pore size of the material. b_2_) The rise length is directly affected by the liquid wetting onto the cellulosic surface, as demonstrated by the imbibition of regenerated cellulose sponges with water and turpentine oil. b_3_) Capillarity and diffusion are shown to be coupled mechanisms during water uptake by porous cellulosic structures. It is shown that after the Jurin height, which is limited by the gravitational force, diffusion plays a dominant role in water uptake. b_1_,b_2_) Adapted with permission.^[^ ^65^ ^]^ Copyright 2018, American Association for the Advancement of Science (AAAS). Reprinted/modified from ref. ^[^ ^65^ ^]^. © The Authors, some rights reserved; exclusive licensee American Association for the Advancement of Science. Distributed under a Creative Commons Attribution NonCommercial License 4.0 (CC BY‐NC) http://creativecommons.org/licenses/by‐nc/4.0/. b_3_) Reproduced under the terms of the CC‐BY Creative Commons Attribution 4.0 International license (https://creativecommons.org/licenses/by/4.0/).^[^ ^66^ ^]^ Copyright 2018, The Authors, Published by Elsevier Ltd.

The singularities observed in the hydration of cellulose arising from the arrangement and organization of its primary forms (polymer, crystal, elementary fibril and bundles) are less pronounced when it comes to larger length scales (dozens of nanometers up to sub‐micrometer scales), where bulk water typically interacts with cellulose constructs of much higher order. At such length scales, wetting arises as a function of the average chemical composition of the exposed surface, as well as its microstructure. Relevant to wetting is the adsorption of hydrophilic molecules (e.g., poly(ethylene glycol) (PEG)) from aqueous solution. For this purpose and to decouple the effect of surface topography, researchers have assumed silica as a model, i.e., used as flat, rigid and perfectly smooth and chemically homogeneous hydrophilic material which displays water wetting similar to cellulose.^[^ ^62^ ^]^ However, special consideration should be given to the effect of roughness associated with the fibril’s natural irregularities and their hierarchical assembly into bundles. In fact, cellulosic surfaces greatly depart from any ideal models. The microstructural features of cellulosic surfaces rapidly convert the hydrophilic character of their inherent chemistry into superhydrophilic (**Figure** **6**a_1_). On the other hand, cellulose can be made hydrophobic or superhydrophobic following functionalization with either bio‐based^[^ ^57^, ^63^ ^]^ or synthetic motifs.^[^ ^64^ ^]^ Such strategies rely on the inherent nanoroughness of the surfaces, mostly comprising cellulose nanofibrils (Figure 6a_2_).

### 5.3 Capillarity and Diffusion

Whereas wetting is tethered to particularities arising from molecular to nanometric length scales, capillary penetration—also termed imbibition or wicking—is intimately linked to the material’s macrostructure. Capillarity and diffusion are key mechanisms for water transport within higher ordered cellulose constructs that are obtained from the respective hydrogels, such as foams (isotropic or directional)^[^ ^65^ ^]^ as well as wood itself, as a source for the development of hydrogels.^[^ ^28^ ^]^ Water interactions and transport at the respective length scale are associated to pores size, their distribution, interconnectivity and hierarchy. Although wicking is at first driven by capillarity, water rising beyond the Jurin height is associated to a Fickian diffusion‐controlled transport.^[^ ^65^, ^66^ ^]^ Both mechanisms are fundamentally coupled to the surface properties of the material as they are affected by variables related to wetting (Figure 6b_1_).

The total capillary penetration scales with the capillary pressure, which is inversely proportional to the pore radius.^[^ ^67^ ^]^ Considering a constant liquid–substrate interface, up to the maximum capillary length, the kinetics of penetration scales with the same proportionality to pore size (Figure 6b_1_). Overall, large pores lead to fast but limited penetration while small ones lead to slow but larger penetration length. For micropores (radius <2 nm) and small mesopores (2 nm < radius < 10 nm) other considerations come into play due to extremely low Reynolds numbers and, thus, highly viscous flows.^[^ ^68^ ^]^ Furthermore, in such circumstances the presence of macromolecules such as proteins or polyelectrolytes,^[^ ^69^ ^]^ e.g., in complex fluids, changes the liquid–substrate interface, consequently affecting the dynamics of the process and the total capillary pressure. For cellulosic substrates, wetting and liquid viscosity promote wicking of a cellulose sponge with water and turpentine oil. It has been demonstrated that the water rise due to capillarity, at the early stages, grows with time (*t*) following *t* ^1/2^ for both liquids (water and turpentine oil); however, at the late stages, the rise height follows *t* ^1/5^ for water and *t* ^1/4^ for the oil (Figure 5b_2_).^[^ ^65^ ^]^ Nevertheless, in general, for an undefined porous material with a known permeability *k*, the Darcy’s law can be used to describe the total water transport, which scales linearly with the capillary pressure normalized by the liquid’s viscosity.^[^ ^70^ ^]^ The relationship between porosity and capillary flow is also described by the Lucas–Washburn equation in which for a given pore radius *r*, the kinetics of imbibition, i.e., the wet distance, grows with time *t* according to ${\left(\frac{\gamma r}{\eta }\right)}^{1/2}t^{1/2}$ with γ being the surface tension of the solvent and η its viscosity.^[^ ^71^, ^72^ ^]^ When the porosity varies in the direction of imbibition, the kinetics is slowed and the time exponent decreases from 1/2 to values depending on various parameters such as pore morphology,^[^ ^73^ ^]^ distribution,^[^ ^74^ ^]^ swelling or elastic response.^[^ ^65^, ^75^, ^76^ ^]^ In the context of soft nanocellulosic materials, the pore size distribution is generally highly polydispersed and, for convenience, the morphology of the pores has been assumed to be circular in cross section. Moreover, the extent of swelling is proportional to the crystallinity of cellulosic material. In such context, the properties (surface tension) of the liquid being infused controls, to some extent, the imbibition rate, and distance, based mostly on wetting constraints.^[^ ^74^ ^]^

As discussed previously, capillarity action determines the early stages of water flow (imbibition) through cellulosic porous material. However, the capillary rise (termed as Jurin height) is limited by the gravitational force. Therefore, any water flow above the Jurin height is attributed to time‐dependent diffusion mechanisms. This has been demonstrated for cellulose sponges by measuring the density of the sponge along the imbibition axis as a function of the imbibition time. It is important to note that the observations made for sponges most likely will not hold for cellulosic foams due to inherent pore characteristic of each material. Whereas sponges are formed by closed cells, foams display interconnected opened cells, which affect strongly capillary forces and imbibition kinetics. The maximum capillary height can be traced at the inflection point (between the time‐independent and time‐dependent density regime in the sponge) (Figure 6b_3_).

For cellulosic systems, capillary flow in sponges made from regenerated cellulose (Viscose) has been extensively studied and used as a model to describe the effects of macromolecular swelling in amorphous cellulosic domains^[^ ^65^ ^]^ and multiscale pore distribution.^[^ ^74^, ^77^ ^]^ The highly amorphous nature of the cellulose in such sponges (≈40% crystallinity)^[^ ^78^ ^]^ leads to various effects such as pore creation and collapse coupled to a swelling, which can be higher than 100%. Furthermore, humidity‐dependent effects are expected for all‐cellulosic systems.^[^ ^79^ ^]^ In the context of nanocelluloses, amorphous domains are considerably less significant with a crystallinity of ≈60% for cellulose nanofibers and up to 90% for cellulose nanocrystals. This makes difficult any comparison of the swelling and the effects inherent to the microstructure due to the different nature of fiber entanglements leading to voids where water interacts with cellulose surfaces. The agreement between predicted wicking kinetics and pore size distribution in sponges made from regenerated cellulose is principally attributed to the dominant role of the macropores in the early stage of wicking and that of sub‐macropores (still micrometer‐scaled) both of which affect the latter stages of diffusion‐controlled water movement (Figure 6b_3_).^[^ ^66^ ^]^ The pore size of the materials (sponges) from which such observations have been made are in a higher length scale of typical nanocelluloses constructs (foams and aerogels), therefore this may not be directly translated but only used as a base for further developments. Additionally, for nanocelluloses, meso and micropores arise from the overlapping regions between nanofibers, leading to complex pore geometries.

The sensitivity to water, a major challenge for the efficient utilization of cellulosic components, needs deep investigation, for example, to unveil the nature and intensity of the involved interactions, especially for the development of moisture responsiveness in multiphase systems and materials.

Transitioning from materials obtained principally by top‐down approaches, including “naturally preserved” wood and wood hydrogels, to materials assembled in a bottom‐up fashion (using building blocks extracted from plants), we note that hydrogels are highly valued given the possibility to finely adjust their physical–chemical properties, for example, to suit given uses. There are ample opportunities in the case of cellulose‐based hydrogels, given the available selection of morphologies of the building block, whether CNC, CNF or otherwise. They are also amenable for functionalization, affecting the nature of the junction zones and degree of crosslinking as well as the possibility to incorporate other components. The latter aspect may consider other polymers, bio‐based or not, as well as active compounds, for example, to tailor given macromolecular properties. By now, it is clear that water interactions are paramount in designing multiscaled cellulosic assemblies. How is such knowledge integrated into a product? Indeed, hydrogels are commonly used in foodstuff (food additives and viscosity modifiers), cosmetics, biomedical (tissue engineering, wound dressing, hygienic products), pharmaceutical (drug encapsulation and delivery systems), agricultural and environmental (water retention, pesticide delivery systems, pollutant adsorbents) fields. Thus, the opportunities for nanocelluloses in these areas are countless. For this reason, and because such subjects have been reviewed, the next section focuses on some enabling processing routes that we consider relevant for product development, from spinning to biofabrication.

### 5.4 Hydrogel Processing Techniques

Once extracted from raw biomass such as wood or other plants, bio‐based colloids are suitably processed into functional materials, with optimized performance. The latter can be reached by design, through the incorporation of multiscaled architectural order and gradients of supramolecular interactions. Several means to assemble biocolloids have, and are currently considered, which are introduced in this section. Historically, the self‐assembly of biopolymers, biocolloids and fatty acids date back to preindustrialization, while regeneration of cellulose was used by the end of the 19th century. These “old” approaches are still heavily investigated using modern techniques. An example is the burgeoning 3D printing, which brings new perspectives to engineer bio‐based materials, using customized and locally sourced building blocks. Alternatively, more exotic approaches associated with the booming of biotechnology in the last three decades have involved synthetic biology and microbial production of biocolloids and their uses. Some technologically relevant processes are presented next.

### 5.5 Spinning

**Figure 7:**
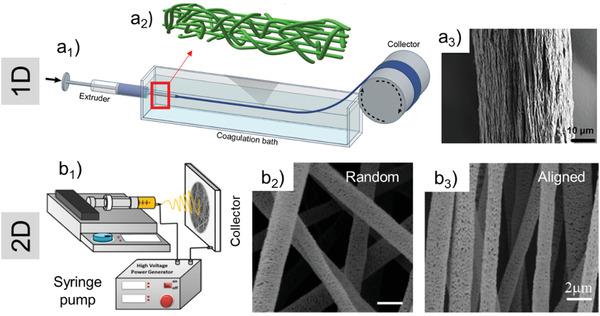
Implementation of spinning processes to obtain 1D and 2D structures from cellulose hydrogels. a_1_) Schematic illustration of a wet‐spinning setup, where a hydrogel is extruded in a coagulation bath and collected as filament. a_2_) Details of nanocellulose alignment where the inner region of a filament is better aligned due to extensional flow while the periphery shows less aligned structures. a_3_) 1D filament with aligned nanofibrils that exhibit about double stiffness and yield strength compared with nonaligned filaments. b_1_) Electrospinning setup where an electric force is used to draw charged threads of a polymer solution. b_2_) Isotropically oriented, and b_3_) aligned PLA/CNC fibrils from electrospinning. a_1_) Reproduced under the terms of the CC‐BY Creative Commons Attribution 4.0 International license (http://creativecommons.org/licenses/by/4.0/).^[^ ^90^ ^]^ Copyright 2019, The Authors, published by Springer Nature. a_2_) Reproduced under the terms of the CC‐BY Creative Commons Attribution 4.0 International license (http://creativecommons.org/licenses/by/4.0/).^[^ ^89^ ^]^ Copyright 2016, The Authors, published by Springer Nature. a_3_) Reproduced under the terms of the CC‐BY Creative Commons Attribution 4.0 International license (http://creativecommons.org/licenses/by/4.0/).^[^ ^91^ ^]^ Copyright 2017, The Authors, published by Springer Nature. b_1_) Reproduced with permission.^[^ ^88^ ^]^ Copyright 2015, Elsevier. b_2_) Reproduced with permission.^[^ ^87^ ^]^ Copyright 2018, American Chemical Society.

Dissolved polymers (wood‐derived or not) are employed in the spinning processes to continuously generate fibers (diameter from <100 nm to several micrometers) that are deposited as a single filament, web (woven) or nonwoven in dry (dry spinning and electrospinning) or wet conditions (wet spinning). In such processes, water interactions are paramount and aspects such as solubility, discussed previously, are of the highest importance. The same processes apply to hydrogels where water interactions affect the rheology, also critically important in extrusion. High molecular weight soluble polymers are suitable for electrospinning, while, high viscosity and shear thinning behavior, are essential for wet spinning of hydrogels.^[^ ^80^ ^]^ Biomimetic nanocomposites based on nanocellulose have been developed through spinning techniques for bone, tissue engineering, and energy storage applications.^[^ ^81^, ^82^, ^83^ ^]^ Cellulose‐based pH‐ and temperature‐responsive hydrogels as well as highly aligned nanocomposites based on CNCs with enhanced anisotropic properties are some examples that benefit from electrospinning.^[^ ^84^, ^85^, ^86^ ^]^ Alignment of nanofibrils is essential to achieve new property spaces from the precursor hydrogels. Highly aligned morphologies are achieved through control on capillary length and diameter during extrusion of 1D filaments via wet spinning (**Figure** **7**a_1_), or by secondary stretching through high speed collection in 2D electrospun mats (Figure 7b_1_).^[^ ^87^, ^88^ ^]^ In fact, as presented in Figure 7a_2_, the traces of orientation caused by extensional flow more effectively aligns the nanofibrils inside the filament, while high shear in the periphery result in a less alignment.^[^ ^89^, ^90^ ^]^ For 1D filaments, long and thin extrusion capillary and high spinning rate orients the nanofibers prior to reaching the coagulant. The increased nanofibril alignment leads to an increased stiffness (19–20 GPa) as well as tensile strength (265–328 MPa), about 50% higher than values reported from nonaligned filaments (Figure 7a_3_).^[^ ^91^ ^]^ The core/shell wet spinning process enables the better alignment of nanocellulose. This method utilizes a supporting polymer in the shell structure that can allow higher spinning rates (up to 33 m min^−1^) while CNF orients in the core.^[^ ^92^ ^]^

The high water content of CNF hydrogels and the low spinning rates (2−13 m min^−1^) may be a drawback in upscaling conventional spinning.^[^ ^92^, ^93^ ^]^ Compared to the sole use of CNF, the concentration of the nanocellulose dope can be increased up to five times by using CNC, obtaining aligned fibers with lower strength and similar stiffness. Furthermore, the availability of CNC in the form of a dry powder is associated with low transportation costs and feasibility in industrial implementation of CNC‐enhanced nanocomposites, to tune the surface structure and mechanical properties.^[^ ^93^, ^94^ ^]^ Figure 7b_2_,b_3_ represent examples of random and aligned poly(lactic acid) (PLA)/CNC nanocomposite mats with up to 20 wt% CNC loading level. By increasing the CNC loading, fibrils display surfaces with higher porosity and tunable tensile properties, based on isotropically oriented or aligned structures.^[^ ^87^ ^]^

### 5.6 3D Printing of Nanocellulose Hydrogels

**Figure 8:**
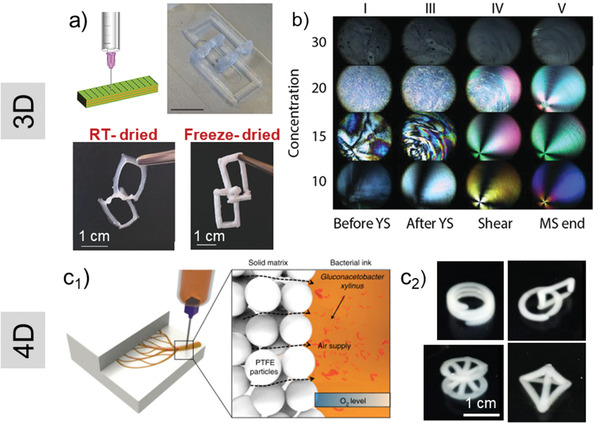
3D printed nanocellulose hydrogel by using the direct ink‐writing technique. a) Comparison of shrinkage extent in the 3D printed sample after drying at room temperature (RT‐ dried) and by freeze drying. Thin films are essentially formed in samples dried at room temperature. Reproduced with permission.^[^ ^101^ ^]^ Copyright 2016, Wiley‐VCH. CNC exhibits color according to its alignment when observed between two cross‐polarized filters. b) Alignment of a CNC hydrogel at different concentrations and shear‐stress observed under optical microscopy between crosspolarizers. Reproduced with permission.^[^ ^104^ ^]^ Copyright 2018, American Chemical Society. Solid Matrix Assisted Printing to develop support‐free complex geometries. c_1_) 3D printing of bacteria‐containing nanocellulose ink inside a PTFE solid matrix. The bacteria were metabolized by access to oxygen supply through solid PTFE particles. c_2_) Development of complex structures such as coil, connected rings, tetrahedron, stacked lattice, and sandglass structures of bacteria‐containing nanocellulose after incubation as printed according to 3D models. c) Reproduced under the terms of the CC‐BY Creative Commons Attribution 4.0 International license (http://creativecommons.org/licenses/by/4.0/).^[^ ^109^ ^]^ Copyright 2019, The Authors, published by Springer Nature.

While most common processing techniques using hydrogels lack sufficient control of the morphological organization,^[^ ^95^ ^]^ 3D and bioprinting have become versatile methods in fields such as tissue engineering and regenerative medicine.^[^ ^96^, ^97^ ^]^ Several 3D printing techniques have incorporated biomaterials in the form of powders, particles, hydrogels and hybrid materials, for example, to fabricate multilayered objects at diverse scales.^[^ ^98^, ^99^, ^100^ ^]^ 3D printing has opened new avenues for constructing bioinspired structures and reproducible tissue mimetics. However, several challenges remain, including optimizing the nature of the printing biomaterial, which need to match the processing method, shrinkage, and the stability of the obtained designs. For instance, because of the high water content and the strong interactions with nanocellulose, hydrogels may collapse upon drying.^[^ ^101^ ^]^ Different drying methods have been studied to preserve the structure of 3D printed architecture; however, the more successful methods, such as freeze drying, are more difficult for scaleup and are more costly. During freeze drying, ice crystals are formed and removed during sublimation through vacuum, and pores are formed to retain the geometry imposed by the ice crystals.^[^ ^5^ ^]^ On the other hand, drying at room temperature is followed by extensive shrinkage even after applying post treatments in coagulation baths (**Figure** **8**a).^[^ ^101^ ^]^ To address these challenges, the solid content of 3D printable hydrogels can be increased by addition of fillers, or anisotropic reinforcing materials such as CNCs. Addition of fillers have a clear effect in the viscosity and printability of the material and sets a limit for viability.^[^ ^102^ ^]^

In 3D printing, shear alignment form percolating networks below a yield stress (nonflowing ink). Partial alignment of particles above the yield stress causes a strong shear thinning response. CNCs, as optically anisotropic particles, exhibit different colors when detected between two crosspolarized filters, depending on their relative orientation. As shown in Figure 8b, at 10 wt% CNC concentration, large domains with similar alignment are visible but as the concentration increases, the aligned domains become smaller due to smaller interparticle spacing, until they are not easily visible anymore.^[^ ^103^, ^104^ ^]^ The shear‐induced alignment of hydrogels (cellulose nanofibrils and nanocrystals) is effective in developing patternable materials with stimuli‐responsive architectures.^[^ ^105^, ^106^, ^107^, ^108^ ^]^ The conventional 3D printing technology can be developed further by introducing Solid Matrix Assisted Printing (SMAP) to fabricate even more complex, support‐free architectures and to overcome the challenge of deformation of 3D printed gels due to gravity, flattening in contact with the solid substrate, and generating structures that are oxygen permeable, critical for aerobic biosynthesis.^[^ ^109^ ^]^ As displayed in Figure 8c_1_, bacteria‐containing nanocellulose ink is extruded in solid matrix of poly(tetrafluoroethylene) (PTFE) microparticles to form complex structures with high control over dimensional stability and topology (Figure 8c_2_).

### 5.7 Regeneration and Coagulation

Besides the methods described so far, regeneration and coagulation are also relevant to the fabrication of structures with controlled properties. At some point of such process, a hydrogel is formed from a dissolution via phase separation and gelation followed by extraction and solvent removal.^[^ ^110^ ^]^ Regeneration is affected by the pH of the precursor solution in association with the isoelectric point of the biopolymer. It is also critically affected by the type and concentration of ions, temperature, and overall, the ionic and electrostatic interactions.^[^ ^111^ ^]^ High molecular mass polymer solutions have more tendency to phase separate due to a less favorable entropy of mixing.^[^ ^112^ ^]^ The reduced number of solvents capable to dissolve cellulose sets a limit in this process. Regeneration of cellulose can be performed in a variety of solutions including water, sulfuric acid,^[^ ^113^ ^]^ and alcohols.^[^ ^114^, ^115^ ^]^ Also, cellulose forms complexes with metal ions. For example, cellulose precipitates when forming complexes with cupper (cuprammonium hydroxide and cupriethylenediamine) or with cadmium (Cadoxen).^[^ ^116^, ^117^, ^118^ ^]^ In addition to alkaline solutions, concentrated aqueous acid solutions such as sulfuric acid, nitric acid and phosphoric acid as well as base solutions like potassium and hydrazine can be employed to dissolve cellulose. Highly strong salt compounds such as sodium iodides and zinc chlorides show promise as cellulose solvents.^[^ ^119^, ^120^, ^121^ ^]^ Swelling and dissolution of cellulose has been reported with tertiary amine oxides. These solvents do not degrade cellulose while having a low toxicity and good solvation power.^[^ ^122^, ^123^ ^]^ Cellulose can be dissolved directly in some ionic liquids (ILs) where the extent of solubility greatly depends on the size and polarization of the anions as well as nature of cations in the IL.^[^ ^124^, ^125^, ^126^ ^]^ Porous cellulose morphologies, including membranes and 3D structures, are produced through phase inversion. The contact of cellulose solvent to an antisolvent creates a thermodynamic miscibility gap that results in the formation of a cellulose‐rich (matrix) and cellulose‐lean (pores) phases. The interpretation of pore formation should consider time and kinetic resolution of the coagulation and during pore generation.^[^ ^127^ ^]^

### 5.8 Multicomponent Compounding

For many hydrogels, the synergy between two or more components is exploited to maximize a given property. Such hydrogels are the subject of many reviews.^[^ ^27^, ^128^ ^]^ Here, we note key examples that incorporate (nano)celluloses and synthetically self‐assembled, crosslinked or gelled materials, for example, toward tissue‐inspired architectures. These cover specific ways to tether water interactions to achieve nature‐inspired, multiscaled structures. As such, multimaterial assembly of nanocelluloses with other components, such as surfactants, noninteracting polymers, oppositely charged (bio)polymers and others, enables supramolecular hydrogel structures, furthering the possibilities as versatile platforms for creating or templating multifunctional materials. For versatility, nanocellulosic hydrogels can benefit from the addition of surfactants that induce secondary assembly.^[^ ^129^, ^130^, ^131^ ^]^ Surfactant addition below a critical mass concentration increases the gel’s modulus while retaining its optical clarity. On the contrary, significant fibril aggregation leads to the loss of optical clarity and visible aggregation is induced over the critical concentration, the value of which depends on the surfactant type. This fact indicates that gel formation is driven by micelle–nanofibril bridging mediated by associative interactions, for example, between the ethoxylated headgroups of a nonionic surfactant and the cellulosic fibrils. Understanding surfactant‐induced nanocellulose assembly will stimulate the development of composite or hybrid nanocellulosic hydrogels.

The assembly of nanocelluloses induced by noncovalent interactions with polymers has been reported.^[^ ^70^, ^71^ ^]^ Inspired from the plant cell structure, wherein cellulose microfibrils are physically assembled together with “soft” hemicellulose chains,^[^ ^132^ ^]^ a biomimetic strategy has been realized by the combination of methylcellulose (MC) with CNC, i.e., to create all‐cellulose thermoreversible and tunable hydrogels.^[^ ^133^ ^]^ As such water‐soluble cellulose derivatives may enhance further the properties of the hydrogels. For example, CNC suspensions, which are liquid at concentrations below 3.5 wt% become viscoelastic at relatively low temperature by the addition of MC. At 60 °C, a strong and distinct hydrogel is obtained at such composition. This tunability results from the interaction of MC and CNC involving physical crosslinks, which accordingly induces the assembly of CNC in a MC network. Simultaneously, the thermoresponsive nature of MC endows such connection with a thermally reversible behavior.^[^ ^134^ ^]^ In a recent work, a green and efficient approach was proposed to create tunable nanocellulose‐based composite hydrogels by incorporating alginate and divalent ions.^[^ ^135^ ^]^ The assembly of cellulose nanofibers and molecular chains of alginate was strengthened by the divalent ions using noncovalent chemistry, which presented a simple and controllable way for composite hydrogel formation. Particularly, it is possible to control the internal structure of the composite hydrogels by the ionic strength of the hydrogel. Owing to the superior performance of such composite hydrogels, a variety of functional applications were tested, including shape‐changeable (3D printing), conductive, and ambient‐dryable porous, light‐weight materials. In a similar report, composite hydrogels comprising CNF and alginate were developed for biomimicking cartilage, wherein CNF simulated the bulk collagen matrix while alginate the proteoglycans. The formed CNF/alginate composite hydrogel was more effective in cartilage growth compared to CNC/hyaluronic acid.^[^ ^136^, ^137^ ^]^ Such assemble approach of functional macromolecules with nanocelluloses hydrogels offers a simple and efficient way toward multifunctional systems.

On similar veins, the self‐assembly of negatively charged nanocellulose and positively charged nanochitin have been used to produce versatile biohybrid hydrogels.^[^ ^138^ ^]^ The self‐assembly process to generate 3D hydrogels was driven by physical crosslinking of CNF and deacetylated chitin nanofibers, via electrostatic interaction and hydrogen bonding. Owing to the rod‐like nature and noncovalent interaction of the building blocks in the hydrogels, a highly interconnected structure was achieved, which ensured superior potential for applications demanding biocompatibility, nontoxicity, and renewability. Other composites based on cellulose and chitin have been inspired by bone microstructure via addition of chitin nanofibers following a templating and reverse‐templating approaches; this method is also scalable to soft tissues.^[^ ^138^ ^]^ Besides oppositely charged materials,^[^ ^139^ ^]^ cationic biopolymers have been used in compounding nanocellulose‐based hydrogels.^[^ ^140^ ^]^ Suspensions of dilute nanocelluloses dispersed in chitosan solution were studied for injection and in situ gelation.^[^ ^141^, ^142^ ^]^ Factors such as hydrogen bonding and hydrophobic interactions were adjusted to optimize the gelation time and properties of the composite hydrogel. The presence of nanocellulose in solutions of chitosan effectively modified their rheological behavior and facilitated hydrogels with self‐healing properties.

Compounding biocolloidal hydrogels, using modern formulation engineering, has enabled applications in drug delivery and tissue engineering. However, several challenges remain, including the design of bioactive hydrogels with suitable biocompatibility, stability, mechanical strength and ability to support nutrition transfer and growth factor delivery. Injectable hydrogels based on cellulose could encapsulate micelles and perform as a secondary barrier for drug release.^[^ ^143^ ^]^ The potential of all‐CNC hydrogels was investigated as injectable drug carrier and sustainable delivery applications. Drug size, solubility, interaction with CNC are determining factors, while the concentration of CNC was found to have a negligible effect on the kinetics of release.^[^ ^144^ ^]^ CNC loading in injectable hydrogels enhances cell adhesion and proliferation but has negligible effect on protein adsorption.^[^ ^145^ ^]^

Several reports discuss physically crosslinked hydrogels by freezing and thawing of aqueous colloidal suspensions, for example, to achieve low density and highly porous structures (porosity over 99.5%).^[^ ^146^, ^147^ ^]^ Upon drying, the water absorption capability and pore size of the obtained aerogels are strongly affected by the freezing temperature and time. This effect is explained by the reverse relationship between degree of supercooling and ice nuclei size, whereby smaller nuclei are formed by faster supercooling, while slower supercooling facilitates the formation of wider pore size range.^[^ ^148^ ^]^

Some hydrogels obtained by conventional methods, such as those involving chemical reactions between monomers and crosslinkers may be weak (stiffness <10 kPa) and typically display toughness and tensile strength of <100 J m^−2^ and <100 kPa, respectively. These values might not sufficiently fulfill the mechanical performance required in applications such as those related to bone and cartilage systems.^[^ ^149^, ^150^ ^]^

Double network hydrogels have been introduced to develop soft and tough structures possessing extraordinary properties as far as strength and toughness and to address the challenges associated with the poor mechanical properties of hydrogels produced by conventional crosslinking. The unique behavior of double network hydrogels is due to the presence of strong interpenetrating structural entanglement, systematic energy dissipation, and a local yielding mechanism.^[^ ^149^, ^151^, ^152^ ^]^ Extending noninteracting nanocellulose and polymers, double network hydrogels comprising gelatin, alginate and CNC have been developed, displaying high mechanical strength (≈14 MPa), and elastic modulus (0.5 GPa) for cartilage applications.^[^ ^153^ ^]^ Particularly, the mechanical properties of a nanocellulose resourced from bacteria (bacterial nanocellulose, BNC) has been found to endow tough ligaments (tensile strength up to 40 MPa) when crosslinked with polyacrylamide, e.g., in the form of double networks.^[^ ^154^ ^]^ Bio‐inspired BNC‐enhanced double network hydrogels comprising lysine, γ‐glutamic acid, and alginate showed a compression modulus of 0.322 MPa, comparable to that of natural articular cartilage. The swelling ratio of BNC was lowered by applying the double network technique and crosslinking to match osteochondral tissue.^[^ ^155^ ^]^ BNC‐gelatin/hydroxyapatite (HA) organic–inorganic hydrogels have been suggested for bone scaffold and biomedical membranes.^[^ ^156^ ^]^ While not the focus of this review, BNC was presented here since it shares quite close similarity to cellulose nanofibrils produced from plants. BNC‐based hydrogels are the subject of extensive investigation and, particularly, they can offer excellent prospects considering the emerging field of biofabrication. For this reason, we spare the next section to discuss such topic.

### 5.9 Biofabrication Using BNC Hydrogels

Although not obtained from wood, gels produced by microorganisms such as bacterial nanocellulose (BNC), share many of the features of the wood‐based counterparts. Nanocellulose biofilms are produced by bacteria onto a variety of fruits, principally tree‐based fruits.^[^ ^157^ ^]^ The biofilms consist of a highly crosslinked pellicle of nanofibers (cellulose I polymorph). Upon removal of the protein component, which is easily conducted by alkaline washing, pure nanocellulose is obtained. This is contrary to the case of wood where lignin or hemicelluloses are present.^[^ ^158^, ^159^ ^]^ The hydrogels of BNC possess a high toughness, water content ≥99%, and maintain their dimensions in water due to the biosynthetic process resulting in a highly interconnected network. Relevant to these hydrogels are the interactions with water, which also applies to all hydrogels discussed so far. This is most relevant to the changes that occur upon drying the hydrogel and subsequent rewetting. Work reporting related effects indicate major differences in water retention, swelling ability and the gain of water upon rehydration. Such phenomena are closely related to the charge density of the respective nanocellulose and the nature of the charges as well as the counterions present in the medium.^[^ ^160^, ^161^, ^162^, ^163^ ^]^

**Figure 9:**
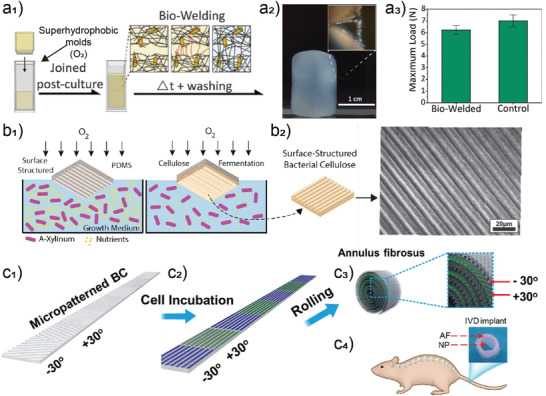
Hydrogels based on bacterial nanocellulose (BNC) can be developed into biofunctional materials. a_1_) Example of living material self‐healing by biofabrication onto superhydrophobic molds, a_2_) image of the bio‐welded structure, and a_3_) tensile properties of the bio‐welded structure compared with a uniform material of similar dimensions. a) Adapted with permission.^[^ ^23^ ^]^ Copyright 2018, Royal Society of Chemistry. b_1_) General templating procedure used for the generation of patterned cellulosic structures by biofabrication. Adapted with permission.^[^ ^24^ ^]^ Copyright 2015, American Chemical Society. b_2_) Example of patterned sheets as obtained after biofabrication. Adapted with permission.^[^ ^168^ ^]^ Copyright 2016, Wiley‐VCH. c_1_) Micropatterned BNC stripe, c_2_) incubated with two cell strains followed by, c_3_) rolling into intervertebral disks (IVD) implants for c_4_) spine injury recovery. c) Adapted with permission.^[^ ^167^ ^]^ Copyright 2018, Wiley‐VCH.

BNC hydrogels have been extensively studied as cell substrates for regenerative medicine and in biomedical research.^[^ ^164^, ^165^ ^]^ They are highly adhesive to cells yet do not trigger inflammatory response.^[^ ^166^ ^]^ The fibril network is also reminiscent of extracellular matrices, although their mechanical properties differ significantly. BNC can be assembled in three dimensions by exploiting the fact that it forms at oxygen‐rich interfaces and therefore the network can be scaffolded using micrometer‐scaled features, further enabling engineering of tissue structures from the hydrogels.^[^ ^23^, ^24^, ^167^, ^168^, ^169^, ^170^ ^]^ Principally, 1) superhydrophobic surfaces have been used to template BNC biofilms with a lateral resolution of 100 µm (superhydrophobic surfaces),^[^ ^23^ ^]^ while 2) 600 nm was reported using poly(dimethylsiloxanes) (PDMS) molds.^[^ ^24^ ^]^ Superhydrophobic templates were used to biofabricate seamless and hollow objects that could be further self‐sutured by growing BNC, effectively demonstrating bio‐welding from living materials (**Figure** **9**a). PDMS microtemplates have been prepared by microfabrication (soft lithography) with features ranging from 100 nm to 50 µm (Figure 9b) and a threshold of 600 nm has been found to be critical for keeping the fidelity of replication after drying, with lateral features from 2 to 20 µm resulting in partial alignment of the biofabricated nanocelluloses.^[^ ^24^ ^]^ Such features are demonstrated to promote aligned growth of cells^[^ ^24^, ^167^, ^168^ ^]^ to produce neuronal tissues, for instance with the potential to repair or replace injured vertebral disks (Figure 9c).^[^ ^167^ ^]^ Alternatively the microtemplated features were also shown to tether the degree of adhesion of cells.^[^ ^170^ ^]^ Overall, BNC hydrogels are demonstrated as excellent substrates for biomedical applications, hinting similar possibilities for plant‐based nanocelluloses.

BNC hydrogels offer a significant advantage over other nanocellulose‐based hydrogels since the former are tough and strong even in the wet state, which enable the formation of double hydrogels by inclusion of a secondary, network‐forming component, such as gelatin.^[^ ^171^ ^]^ Addition of another polymeric component can lead to double network hydrogels that can mimic the properties of cartilaginous and other soft tissues where a combination of high toughness, and strain recovery can be achieved.^[^ ^152^ ^]^

## 6 Introducing Nanoscaled Anisotropy in Hydrogels

Nanoscaled hierarchical structures with controlled long‐range order are inherent to biological systems, with many natural multiscaled architectures being formed from nanofibrils.^[^ ^172^ ^]^ This section introduces the means by which long‐range order is introduced in nanocellulose hydrogels. The specific advantage of biocolloids to generate long‐range order in hydrogels, when compared to top down processing of natural materials, is the possibility of a high scalability and a more versatile range of morphologies. In contrast, shape and size are limited by the (bulk) organism used in top‐down approaches, as obtained in nature (e.g., wood and crustaceans shells^[^ ^173^ ^]^). Furthermore, bottom‐up fabrication offers the advantage of facile compositing processes, where the formulation can be tuned, which is more easily merged into modern, industrial, manufacturing processes. Besides multiscaled structures that combine nano, micro to macroporous structures, as in wood, long‐range order at the nanoscale from wood‐based nanofibrils, and biocolloids in general, involves principally two ordered states: 1) nematic order, where the fibrils are aligned relative to each other and, 2) chiral nematic order, where the fibrils are aligned relative to each other within a loose plane but rotate relative to each other along a director perpendicular to that plane.

### 6.1 Nematic Ordering

Nematic ordering of nanocellulosic hydrogels, with fibers aligned along a given orientation, has been achieved principally to improve the mechanical strength and toughness of the subsequently dried, nonporous materials, e.g., by improving the supramolecular interactions between nanofibrils. Thereafter, porous materials for thermal insulation can be obtained by supercritical drying. Although nematic order brings critical properties to the hydrogels, such as controlled ionic flow,^[^ ^174^ ^]^ the principal use of hydrogels with controlled orientation is as an intermediate state for the formation of (dry) materials. Except for their use as precursors of aerogels, principally used for thermal insulation, superstructured nanocellulose hydrogels still remain largely underutilized.

Mainly achieved with the less crystalline cellulose nanofibers, the most common route for nematic ordering is solvent exchange or acidification, which induces rapid and unidirectional gelation, for example, of carboxyl functionalized cellulose (e.g., periodate or 2,2,6,6‐tetramethylpiperidine‐1‐oxyl radical (TEMPO)‐oxidized nanocelluloses).^[^ ^175^, ^176^, ^177^ ^]^ As such, partial dewatering generally occurs as a result of reduced water interactions, where the semicompressed nematic gel forms within the solvent exchanged vial. The obtained hydrogels transform into aerogels by supercritical drying and show a remarkably high mechanical strength and toughness compared with their randomly oriented counterparts.^[^ ^2^, ^176^ ^]^ The specific surface area of aerogels can be increased by decreasing the charge density of the precursor cellulose nanofibrils. In turn, loading hydrogels with a drug was shown to occur at higher rates in hydrogels of low surface area that resulted from nanofibrils of high charge density. The release rate was proportionally slower for higher loading rates, where the nematic ordering is expected to play a role in the directional release of given pharmaceuticals.^[^ ^178^ ^]^

**Figure 10:**
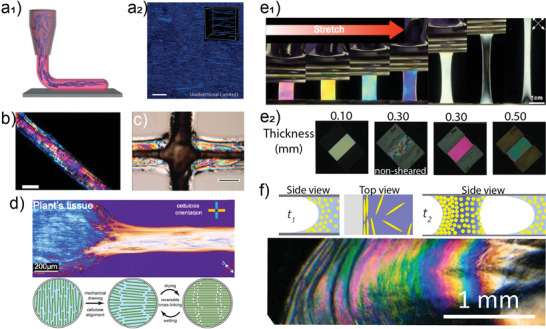
Nematically aligned nanocellulosic hydrogels and their resulting materials. The images were obtained between crossed polarizers, where color changes are associated with changes in relative orientation of the fibrils. a_1_) 3D printing of microfibrillated cellulose with a_2_) millimeter‐sized long‐axis scale fiber ordered on the macroscale. The scale bar in (a_2_) is 200 μm. a) Reproduced with permission.^[^ ^108^ ^]^ Copyright 2016, Springer Nature. b) Alignment in wet‐spun TEMPO‐oxidized CNF, and c) in 3D‐printed CNC filaments with retardation colors from aligned fibers as observed between crossed polarizers. The scale bar in (b) is 50 μm and the scale bar in (c) is 200 μm. b) Adapted under the terms of the CC‐BY Creative Commons Attribution 4.0 International license (http://creativecommons.org/licenses/by/4.0/).^[^ ^91^ ^]^ Copyright 2017, The Authors, published by Springer Nature. c) Adapted with permission.^[^ ^185^ ^]^ Copyright 2017, Wiley‐VCH. d) Mistletoe berry filaments obtained by stretching hydrogels present in the fruit tissue as observed between polarizers, the filament behaves as a regular CNF‐spun film, as described schematically in the bottom panels. Adapted with permission.^[^ ^202^ ^]^ Copyright 2019, American Chemical Society. e_1_) Surface shear induced alignment in hydrogel films of CNCs as a function of thickness of the hydrogel layer, nonsheared sample (UH4) is presented on the right‐hand side. Adapted with permission.^[^ ^189^ ^]^ Copyright 2018, Royal Society of Chemistry. e_2_) Elastomeric compound obtained from the precursor hydrogel film and its retardation colors as observed as a function of stretching between polarizers. Adapted with permission.^[^ ^188^ ^]^ Copyright 2019, American Chemical Society. f) Example of confinement‐induced nematic order as reached during evaporation of CNC dispersions between substrates, with decreasing birefringence from the outer edge (left) to the center of the wet area (right). Reproduced with permission.^[^ ^204^ ^]^ Copyright 2020, Wiley‐VCH.

The other main approach to induce nematic orientation in hydrogels exploits the hydrodynamics of the dispersions, for example, when shear forces are applied onto the gelling or gelled network of nanofibrils. In this context, filaments are produced with aligned nanocellulose under the shear by wet^[^ ^89^, ^91^ ^]^ or dry^[^ ^179^, ^180^ ^]^ spinning, or by applying external shear.^[^ ^181^ ^]^ Drawing filaments under controlled conditions (**Figure** **10**b) has enabled strong and stiff materials from nanocellulose, with strengths at break above 1 GPa and stiffness above 50 GPa.^[^ ^182^, ^183^, ^184^ ^]^ This strategy is also utilized in 3D printing or in microextrusion, where nanocrystal alignment of up to 86% is possible in the wet state (Figure 10a,c).^[^ ^104^, ^185^, ^186^ ^]^ Given the 3D, predictable response, such alignment could be used to control the humidity and thermal response of nematically oriented filaments.^[^ ^108^ ^]^ In the case of films, the shear‐aligned hydrogels have been obtained from shearing glucose‐infiltrated gels and used to control the optical properties of elastomeric matrices (polyacrylate) (Figure 10e).^[^ ^187^, ^188^, ^189^ ^]^

Another mechanically induced approach to increase the relative alignment of fibers is wet stretching of the obtained gels. Interestingly, chiral nematically ordered films can be stretched to unwind the chiral‐nematic director into a purely nematic order.^[^ ^187^, ^190^ ^]^ Chiral nematic hydrogels can also be effectively aligned by using a strong electric field.^[^ ^191^ ^]^ Alternatively, BNC sheets could be stretched to align the fibrils into high strength filaments and films. It has been reported that wet drawing (by 30% to 40%) results in a highly aligned fibrils (and tensile strengths of the obtained material of up to 1 GPa). In comparison, stretching of cellulose nanofiber hydrogels results in a tensile strength of 0.4 GPa.^[^ ^192^, ^193^, ^194^ ^]^ In a similar manner, the drying stresses that develop under geometrical constraint leads to the alignment of cellulose nanofibrils, cellulose nanocrystals, and dissolved cellulose gels.^[^ ^195^, ^196^, ^197^, ^198^ ^]^

Drying nanocellulose fibers in the presence of alginate followed by reswelling has been used as an alternative method to achieve high fibril alignment.^[^ ^199^ ^]^ Other routes for inducing nematic order are possible, for example, by solution processing of nanocellulose dispersions, where the physicochemical interactions induce alignment. For instance, osmotic dehydration resulted in high volume fraction (up to 4.9%) of aligned (order parameter of ≈0.8) CNF.^[^ ^200^ ^]^ Other approaches with CNC in the presence of a suitable electrolyte and concentration indicate the possibility of an arrested state before chiral‐nematic order sets in the gelled state.^[^ ^201^ ^]^ Alternatively, during freeze casting, ice growth in aqueous dispersions of CNF resulted in fibril alignment within the walls separating the growing ice.^[^ ^40^ ^]^ Another more exotic route is the use of raw biomass (mistletoe berry), with minimal processing, to obtain nematically oriented filaments (Figure 10d).^[^ ^202^ ^]^ Hydrogels directly obtained from berries could be wet spun into strong filaments by aligning the cellulosic fibers contained in the soft fruit. In another implementation, spheroidal nanoparticles extracted from regenerated cellulose (i.e., cellulose II polymorph) were demonstrated to self‐assemble into highly aligned nanofibrils in the hydrogel state, thus yielding films with a high transparency.^[^ ^203^ ^]^ In another report, capillary forces as obtained under confinement between two substrates, were shown to induce alignments in gelled dispersions undergoing evaporation.^[^ ^204^ ^]^ The different processes reported to align nanocelluloses hints at the necessity of adapting processes, for future large‐scale operations, to the specific building blocks (e.g., nanocrystals or nanofibrils) in order to tether the opto‐mechanical properties of the obtained materials.

### 6.2 Chiral Nematic Order

**Figure 11:**
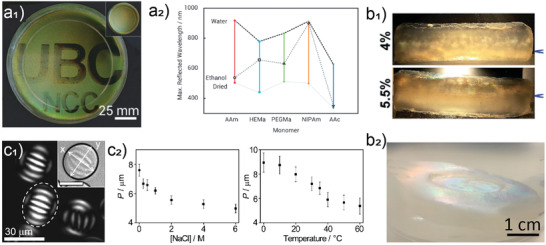
Chiral‐nematically ordered nanocellulosic organogels and hydrogels. a_1_) Chiral‐nematic oriented hydrogel and a_2_) its response as a function of polymeric matrix encasing the chiral‐nematic hydrogels. a) Adapted with permission.^[^ ^212^ ^]^ Copyright 2013, Wiley‐VCH. b_1_) Chiral‐nematic organogels in acetone indicating the isotropic fraction to chiral‐nematic fraction in phase separated and solvent‐exchanged samples. b_2_) Example of iridescence obtained from a 25 wt% organogel. b) Adapted with permission.^[^ ^2^ ^]^ Copyright 2019, Royal Society of Chemistry. c_1_) Microgel obtained from chiral‐nematic phases crosslinked post microfluidization crosslinking. c_2_) Dependence on the dynamic response of the microgels’ features (helicoidal pitch) distances as a function of electrolyte concentration or temperature. c) Adapted with permission.^[^ ^211^ ^]^ Copyright 2016, Wiley‐VCH.

Chiral nematically ordered hydrogels obtained from CNC do not enhance supramolecular interactions as efficiently as those from nematically ordered nanocelluloses due to mismatched alignment across layers. However, stress dissipation in compression and a range of responsive optical properties can be developed.^[^ ^2^, ^205^ ^]^ These two properties are typically exploited in nature to form materials with high fracture resistance and specific nonfading colors.^[^ ^206^, ^207^, ^208^ ^]^ Chiral nematic order occurs due to the lyotropic liquid‐crystal transitions of CNC dispersions above a given threshold, where water interactions are optimized through the phase transitions occurring as water is evaporated or removed. Chiral nematically arranged gels are typically obtained by arresting evaporation at a given concentration, depending on the physicochemical properties of the CNC (**Figure** **11**a).^[^ ^2^, ^209^, ^210^ ^]^ Related approaches include swelling of films formed by evaporation‐induced self‐assembly^[^ ^187^ ^]^ or processing of a fully formed chiral‐nematic phase into emulsions.^[^ ^211^ ^]^

The more common approach for the formation of responsive chiral nematic hydrogels from CNC has been coevaporation of synthetic hydrogel precursors with the colloidal nanocrystals.^[^ ^212^, ^213^ ^]^ Photopolymerization resulted in hydrogels with tunable reflection wavelengths, from 550 to >1100 nm.^[^ ^212^ ^]^ Single‐component gels formed by solvent exchange of phase‐separated liquid crystalline CNC suspensions resulted in well‐defined domains and periodic structures, with structural colors observable in the gels as a function of their density (Figure 11b).^[^ ^2^, ^214^ ^]^ In other efforts, hydrogel microdroplets resulted in stimuli‐responsive microgels. Their helicoidal periodicity varied from 5 to 9 µm with electrolyte concentration (0 to 6 m) or temperature (10 to 50 °C) (Figure 11c). In turn, the effect of confinement resulted in a catalytic activity that was higher than their free counterparts; although stimuli‐response effects were not evaluated.^[^ ^211^ ^]^ Embedded catalytic silver particles were also explored to enhance catalytic activity of the microgels. “Cup”‐shaped chiral nematically arranged microgels were also obtained by compounding droplets of mineral oil with chiral‐nematic ones.^[^ ^215^ ^]^ These efforts highlight that the multiscaled, chiral nematic architectures can be used to engineer hydrogels with a variety of mechanical and optical responses.

## 7 Counterions, Multivalent Cations, and Associated Mineralization

Monovalent and multivalent counterions are known to dynamically affect colloidal interfaces.^[^ ^216^ ^]^ These ions can reduce interfacial hydration layer as well as the length to which water form hydrogen‐bonded networks, from the interface of the biocolloids. As such, they tether the energy potential at the biocolloid/water interphase observed in dispersions. In turn, counterions can lead to the formation of hydrogels or reinforce their mechanical properties. These interactions with nanofibers are ubiquitous in nature, due to the high salinity of biological fluids, and are generally preliminary interactions toward biomineralized constructs. Ion interactions also affect the behavior of hydrogels when introduced in various biological solutions.^[^ ^217^ ^]^ Here, we discuss how the surface chemistry, geometry, and mechanical properties of wood biocolloids impact their interaction with electrolytes, beyond covalent interactions, for the formation of new and improved materials.

For nanocelluloses, the inherent interactions of ions with cellulose is relatively low, however introduction of negative charges (e.g., from sulfate or carboxylate groups) during the extraction process significantly affects their dynamic interactions with mono and multivalent ions. These interactions have been used to control the aggregation of nanocelluloses with various functional groups, leading to a vast set of hydrogels for uses that range from specific metal capture,^[^ ^218^ ^]^ regenerative scaffolds,^[^ ^219^, ^220^ ^]^ and high strength and toughness hydrogels.^[^ ^221^ ^]^

### 7.1 Monovalent Ions

Monovalent ions can be used to tune the phase transition of liquid crystals,^[^ ^201^ ^]^ and to control the aggregation of nanocelluloses, from dispersions to gels;^[^ ^222^ ^]^ furthermore, they can substantially influence the swelling potential of charged gels.^[^ ^216^ ^]^ The interaction of cations with oppositely charged anions varies significantly depending on the chemical accessibility to the anion (steric effects) and polarization of the charged groups. Accordingly, the functional groups, typically, sulfate half‐esters for CNCs and carboxyl for oxidized CNFs, display given interactions due to their surface chemistry, with cations interacting with sulfate preferentially over carboxylate groups.^[^ ^223^, ^224^ ^]^ Other effects, such as increased crowding at lower concentrations for CNFs compared with CNCs, also play a critical role. For instance, CNCs inherently form gels at volume fractions between 3–8% and can be processed at up to 50%.^[^ ^104^ ^]^ In contrast, CNF gels are obtained at or below 2% and are hardly processed at concentrations above 5%.^[^ ^200^ ^]^

**Figure 12:**
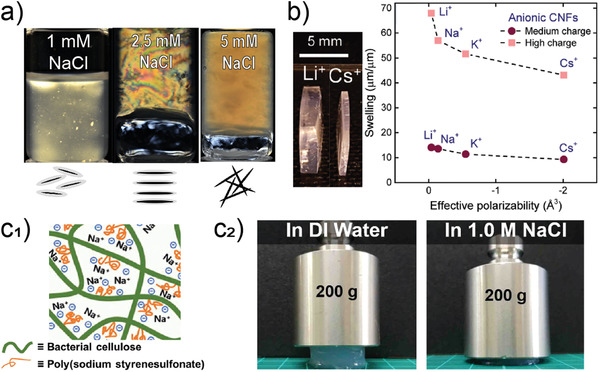
Effect of monovalent ions on the properties of nanocellulose hydrogels. a) Effect of monovalent electrolytes on CNCs hydrogels, where the gel transitions from a repulsive low viscoelasticity gel, to a partially nematically ordered gels to an aggregated gel upon increasing the electrolyte concentration from 1 × 10^−3^ to 5 × 10^−3^ m. Adapted with permission.^[^ ^201^ ^]^ Copyright 2017, American Chemical Society. b) Impact of monovalent counterion on swelling of TEMPO‐oxidized CNFs as a function of the ion polarizability. Adapted with permission.^[^ ^216^ ^]^ Copyright 2019, Royal Society of Chemistry. c) BNC hydrogel response to Na counterion concentration where a significant decrease in compressive strength and water retention is observed upon addition of electrolytes. Adapted with permission.^[^ ^217^ ^]^ Copyright 2018, Royal Society of Chemistry.

Monovalent ions induce gelation principally by screening electrostatic repulsion and promotion of attractive van der Waals forces. This interaction is exploited in nanocellulosic hydrogels infiltrated with anionic polyelectrolytes to result in salt‐dependent strength, with a compressive strength four fold higher in pure water than in the presence of 1 m of NaCl (**Figure** **12**c).^[^ ^217^ ^]^ Hydrogels of CNCs formed by aggregation induced by monovalent ions display a yield stress that is an order of magnitude higher compared to their high concentration counterparts.^[^ ^225^ ^]^ However, intermediate nonaggregated states can promote nematic order in CNC gels (Figure 12a).^[^ ^201^ ^]^ Zwitterionic CNCs, or CNCs with low sulfate substitution aggregate more readily by addition of electrolytes.^[^ ^226^ ^]^ CNC gelation is promoted by Cs at the lowest concentration followed by K, and Na. The highest concentration required to induce aggregation is with Li (Figure 12b).^[^ ^222^ ^]^ The same trend has been observed for the kinetics of swelling of films of carboxylated CNFs into hydrogels, with the largest and least swelling observed for Li and Cs, respectively.^[^ ^216^ ^]^ This suggests that the nature of the cation, e.g., with higher dispersion interactions than ion–ion correlation for Cs, has a stronger impact than the functional anionic group of the nanocelluloses. The ion series did not significantly influence the biodegradation of carboxylated CNFs, suggesting their efficient implementation for disposable applications.^[^ ^227^ ^]^ Swelling of carboxylated CNF films into gels was more pronounced in the presence of acetate, followed by HCl, phosphate, and citrate.^[^ ^216^ ^]^ Both carboxylated CNFs and sulfated CNCs aggregated at similar NaCl concentrations (≈35 × 10^−3^ m NaCl).^[^ ^225^, ^228^ ^]^ Essentially, due to the colloidal nature of nanocelluloses, monovalent ions can efficiently promote their interaction with water.

### 7.2 Multivalent Ions

Compared to the monovalent counterparts, multivalent ions have a more significant impact on nanocellulose gelation in aqueous media, with the required gelation concentration of multivalent cations reduced by at least an order of magnitude per increased ionic charge.^[^ ^222^ ^]^ Additionally, water retention,^[^ ^221^ ^]^ and mechanical robustness^[^ ^227^ ^]^ of the obtained nanocellulose hydrogels can be increased by at least an order of magnitude.^[^ ^221^, ^227^ ^]^

Multivalent ions, generally metals, interact with functional groups on nanocelluloses by coordination, electrostatic interactions, or via double‐layer interactions.^[^ ^216^ ^]^ Many multivalent ions also have chemical or biological functionalities; they are therefore widely evaluated for the formation of nanocellulose hydrogels for biomedical applications. For instance, carboxylated CNF hydrogels crosslinked with calcium have been evaluated favorably in regenerative medicine due to calcium influence on hemostasis and the fate of epidermal cells.^[^ ^219^, ^220^, ^229^ ^]^ The effect of calcium on the mechanical robustness of gels formed from carboxylated CNFs has been noted to be more pronounced than (covalent) glutaraldehyde‐based crosslinking and could thereafter be used to template fibroblasts growth into tubular constructs.^[^ ^219^ ^]^ The specific affinity of nanocellulose hydrogels with multivalent ions has also been considerably explored in the context of metal capture to isolate harmful contaminants from waste effluents. For instance, nanocelluloses were demonstrated to have a high affinity for the capture of a vast range of multivalent ions, with, e.g., 710 mg of mercury adsorbed per gram of amide functionalized CNCs.^[^ ^218^ ^]^ Compared to their sulfated counterparts, phosphorylated CNCs showed substantially higher adsorption capacity for Cu^2+^, Fe^3+^, and U.^[^ ^230^, ^231^ ^]^ Mechanically fibrillated nanocellulose showed better affinity to Cu^2+^ than sulfated CNCs. The upper performers in terms of metal capture being reported to be phosphorylated CNCs and TEMPO‐oxidized CNFs (or TO‐CNFs).^[^ ^218^ ^]^ Interestingly, CNCs bearing the combination of sulfate and carboxylate are reported to have a higher affinity than sulfated or carboxylated CNCs.^[^ ^232^ ^]^

**Figure 13:**
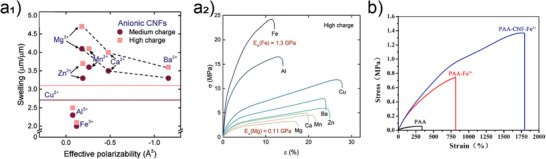
Effect of multivalent ions on the properties of nanocellulose hydrogels. a_1_) Effect of multivalent ions on the swelling of films of TEMPO‐oxidized CNFs into hydrogels. The complex interaction is elucidated for each multivalent counterion (right). Adapted with permission.^[^ ^216^ ^]^ Copyright 2019, Royal Society of Chemistry. a_2_) Wet strength in tension of resulting materials, showing an increase above tenfold, depending on the ion type. Adapted with permission.^[^ ^234^ ^]^ Copyright 2019, Wiley‐VCH. b) Synergistic effects of CNFs and Fe^3+^ on improving the mechanical properties of the composites. Adapted with permission.^[^ ^221^ ^]^ Copyright 2017, American Chemical Society.

CNFs have been investigated more extensively than CNCs for the formation of hydrogels via multivalent interactions. Although multivalent cations can efficiently stabilize CNCs dispersions into hydrogels as well.^[^ ^233^ ^]^ The principal reason behind the deeper exploration of CNFs is related to the correlation between the hydrogel behavior and the wet resistance of the dry materials formed, the latter being heavily explored in terms of the mechanical performance for carboxylated CNF (**Figure** **13**a_2_).^[^ ^216^ ^]^ Swelling of carboxylated CNF films was minimal when crosslinked with Fe^3+^ being the highest valence ion evaluated, closely followed by Al^3+^ (Figure 13a_1_). In turn, the elastic moduli of the formed wet materials increased by more than tenfold for carboxylated CNF crosslinked with Fe^3+^ rather than Mg^2+^.^[^ ^216^ ^]^ Surprisingly, Pb^2+^ showed a better affinity for TO‐CNFs than Al^3+^. The enhanced mechanical attributes of the wet materials were attributed to the stronger interactions by coordination between given metals and CNFs. Similar properties to TEMPO‐oxidized CNF were observed for phosphorylated CNF, In turn, carboxymethylated CNF displayed a significantly higher toughness in the form of wet films, crosslinked with multivalent ions, compared with the corresponding phosphorylated CNF or TO‐CNF.^[^ ^234^ ^]^

The integration of synthetic polymers further widens the property space for hydrogel‐based materials. For example, metal‐ion crosslinked TO‐CNF hydrogels infused with polyacrylamides show good self‐healing and shape recovery after strains as high as 750% (Figure 13b).^[^ ^221^ ^]^ The synergy between the three components is remarkable and further suggestive of the potential of multinetwork hydrogels from nanocelluloses, as previously demonstrated for bacterial cellulose or modified cellulose nanocrystals.^[^ ^235^ ^]^

### 7.3 Mineralization and Biomineralization of Ion‐Infused or Ion‐Crosslinked Nanocellulose Hydrogels

**Figure 14:**
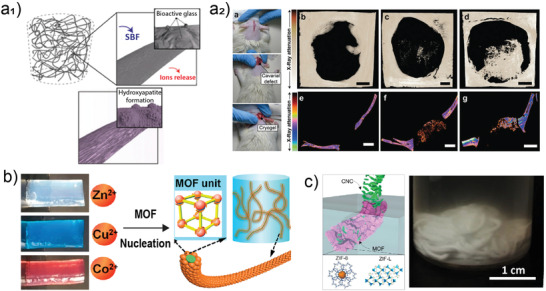
Use of ions as precursor for the formation of mineralized nanocellulose hydrogels. a_1_) Bioglass loaded CNF hydrogels and their mineralization upon ion‐release and interaction with stem cells. a_2_) Cavarial defect regeneration as a function of the gel content after 56 days of implantation ((b–d) and (e–g) in (a_2_) are 3D through and across slices, respectively—the scale bars are 1 mm). a) Adapted with permission.^[^ ^239^ ^]^ Copyright 2019, Royal Society of Chemistry. b) Metal crosslinked gels and their nucleation into MOF‐containing hydrogels. Adapted with permission.^[^ ^237^ ^]^ Copyright 2018, American Chemical Society. c) Continuous nucleation of MOFs into core–shell filaments structure as triggered by the introduction of CNC gels. Adapted with permission.^[^ ^238^ ^]^ Copyright 2019, American Chemical Society.

Mineralization of ion‐infused or ion‐crosslinked nanocellulose hydrogels is one of the more promising approaches to generate composites with advanced properties. Beyond synthetic petrification of wood biomass,^[^ ^236^ ^]^ nanocellulose dispersions and hydrogels have been explored in the context of mineralization for bone regeneration, calcium carbonate mineralization, and the growth of metal–organic frameworks (MOFs). The latter case is an active field of research, where initial gelation of TO‐CNFs is promoted by Zn^2+^, Cu^2+^, and Co^2+^ followed by sequential immersion of the metal‐crosslinked hydrogels in metal and ligand precursors leading to nucleation of MOFs onto the fibrils’ surface (**Figure** **14**b).^[^ ^237^ ^]^ The multiscaled pore structures of the resulting materials enables a remarkably high selective adsorption capacity for model compounds (synthetic dyes). In a different implementation, continuous mineralization was exploited using extrusion of CNCs in a bath containing the respective precursors at a ratio that prevented the formation of large crystals, i.e., with an excess Zn^2+^ (Figure 14c). Continuous mineralization was triggered upon exposure of the precursor solution to the negative charges present on CNCs.^[^ ^238^ ^]^ The extruded filaments were used for encapsulation of a model enzyme, demonstrating the dependence on mineralization time toward their bioactivity.

In the case of bone regeneration, precursors to the generation of HA or osteoblasts mineralization were introduced in nanocellulose gels. While BNC has been heavily investigated in the past decade as bone mineralization substrate, nanocellulose studies used in that context are quite recent (Figure 14).^[^ ^239^ ^]^ Compositing bioglass (Bioglass 45S5 comprising 46.1 mol% SiO_2_, 24.4 mol% Na_2_O, 26.9 mol% CaO, and 2.6 mol% P_2_O_5_) with a CNF network yielded a hydrogel that, when in contact with simulated body fluid, is able to efficiently nucleate the crystallization of HA while releasing essential ions to stimulate the production of key bone morphogenetic proteins from cells. Both aspects resulted in efficient bone growth across induced defects. In the case of calcium carbonate mineralization, an essential part of tough and strong biological architectures,^[^ ^206^ ^]^ several efforts have demonstrated the versatile application of CNCs for its controlled mineralization. Chiral nematically ordered gels (swollen films) could be used to form calcite, mimicking crustaceans architecture, with a chiral nematic architecture.^[^ ^240^ ^]^ In contrast, highly charged “hairy” CNCs, with charges above five times that of regular CNCs were shown to readily inhibit mineralization of calcium carbonates.^[^ ^241^ ^]^ Although current research on mineralization of nanocelluloses indicates exciting prospects, as mineralized fibrous structures are ubiquitous in nature,^[^ ^172^ ^]^ significant progresses paralleling ion interactions studies need to be pursued to enable the formation of high performance biomimetic materials.

**Figure 15:**
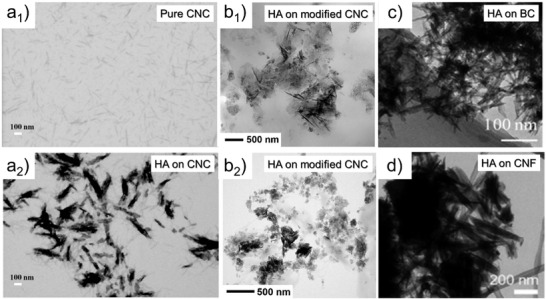
Comparison of HA crystallization on different morphologies of CNC, CNF, and BNC. a_1_) CNC before, and a_2_) after HA mineralization when the HA content is controlled by pH in solution. a) Reproduced with permission.^[^ ^242^ ^]^ Copyright 2019, Royal Society of Chemistry. b_1_) CNC succinic acid carboxylate (CNWSAC) and b_2_) CNC succinic anhydride acetic anhydride ethylenediamine (CNWSAAE) after 14 days of HA mineralization. b) Adapted with permission.^[^ ^245^ ^]^ Copyright 2019, Elsevier. c) Multivalent assembly of nanoscale HA crystal needles using BNC as a reactor for controlled mineralization of HA. Reproduced with permission.^[^ ^243^ ^]^ Copyright 2019, Royal Society of Chemistry. d) Bright‐field TEM image showing randomly oriented mineral crystals on nanoaligned cellulose nanofibers. Reproduced with permission.^[^ ^244^ ^]^ Copyright 2019 American Chemical Society.

Different types of 3D scaffolds have been developed inspired by the intrinsic relationship between human bone and HA to highlight the drawbacks of currently used bone substitutes.^[^ ^84^, ^242^ ^]^ Biomimetic nanocomposites based on cellulose and HA are promising biomaterials for bone tissue engineering. Transmission electron microscopy (TEM) of various nanocellulose substrates for HA crystallization is displayed in **Figure** **15**.^[^ ^242^, ^243^, ^244^, ^245^ ^]^ Huang et al. developed a facile approach (via pH adjustment) to coat HA on CNC in a simulated body fluid. Generally, pH control rather than CNC surface modification is more effective in HA formation (highest HA formation at pH 9) (Figure 15a_1_,a_2_).^[^ ^242^ ^]^ This approach is in contrast to most studies on CNC/HA composite that consider a blend of materials that leads to a heterogeneous mixture with insufficient mechanical properties. In an alternative approach, Fragal et al. developed bioinspired CNC–HA nanocomposite by introducing reactive anionic sulfate and phosphate groups on the surface of CNC and controlling the HA formation on CNC by the carboxylate and amine group content.^[^ ^245^ ^]^ Figure 15b_1_,b_2_ display similar effectiveness of sulfate and phosphate reactive groups in the first 14 days of HA synthesis. Also, as shown in the TEM image of Figure 15c, BNC proved to perform as an effective assembly reactor with potential to orient HA crystallization through controlling the phosphate mineralization process. According to the mineralization mechanism, the driving factors in the assembly of HA are the weak coordination of hydroxyl groups in nanocellulose with calcium ion and the steric hindrance in the hydrogel structure.^[^ ^243^ ^]^ Also, hybrid nanocomposite hydrogels with nanostructural features were developed by mineralization of HA on highly compact and well aligned CNF (Figure 15d). Poly(acrylic acid) was utilized as a mineralizing agent for TEMPO‐oxidized CNF and the highly mineralized hydrogel. The mineral content and the mechanical properties of the nano‐aligned hydrogel resembles those of natural hard tissue, including the human dentin and mouse cortical bone.^[^ ^244^ ^]^

## 8 Nature‐Inspired Hydrogels

**Figure 16:**
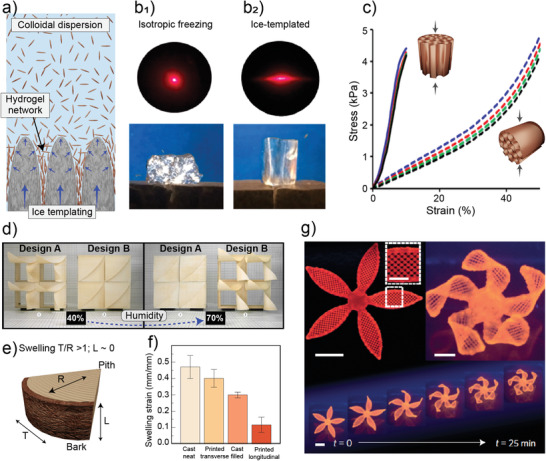
Nature‐inspired hydrogels. a) Ice‐templating of colloidal suspensions leading to directional pores and aligned nanoelements, which replicates the wood ultrastructure. b1,b2) Comparison of the cellulose biocolloids alignment obtained from isotropic freezing (b_1_) and ice templating (b_2_). a,b) Adapted with permission.^[^ ^40^ ^]^ Copyright 2016, American Chemical Society. c) Templated CNC hydrogels typically display anisotropic mechanical behavior when stressed at the parallel or perpendicular direction in relation to the aligned pores; however, the hydrogels are tougher at the perpendicular direction. Adapted with permission.^[^ ^250^ ^]^ Copyright 2016. American Chemical Society. d) Computer‐designed complex wood laminar structures that explore the e) wood inherent swelling/shrinkage anisotropy to develop humidity‐driven actuators. Adapted with permission.^[^ ^254^ ^]^ Copyright 2015, Elsevier. f,g) Versatile inks based on CNFs can be prepared to display anisotropic swelling (f), and therefore to yield man‐made complex 3D structures that have high responsiveness to humidity (g). Adapted with permission.^[^ ^108^ ^]^ Copyright 2016, Springer Nature.

Research efforts in mimetism have taken inspiration from vascular plants, where the multiscaled structures, particularly present in wood, are used for the preparation of colloidal hydrogels that display anisotropic mechanical performance and responsive spatial actuation over external stimuli. The microstructure of wood has been replicated in man‐made hydrogels, mostly through ice templating. Hydrogels with highly aligned cellulosic colloids forming tubular, vascular‐like structures are achieved by controlling the phase change of liquid water into ice crystals. In this process, the relative amount of bound, freezing and free water in the biocolloid suspension play an important role, e.g., in the phase change of liquid water adjacent to the colloids into ice crystals, which is especially relevant for suspensions containing high solid fractions. These topics have not been systematically discussed in the recent literature. In a typical ice templating technique, the freeze‐thaw process, a colloidal suspension (CNFs or CNCs in the context of this review) is subjected to a directional temperature gradient where the growth of ice crystals takes place in one side of the suspension and grows along the temperature gradient. Depending on the cooling rate, the colloids can be: i) pushed ahead as the ice front moves upward, ii) templated, or iii) get entrapped in the ice. Thus, with an optimized cooling rate, lamellar ice crystals form and do not accommodate the colloids within their lattice, leading to a redistribution of the suspended particles in between the templating ice crystals (**Figure** **16**a). This causes a local increase in solid fraction that results in aggregation of the colloids and network formation. Cellulose biocolloids are especially attractive in the context of such technique. Nanocelluloses’ high aspect ratio (≈50) enables highly entangled networks at very low solid fraction (<2 wt%), thus achieving strong hydrogels with very high water content. Although single step ice templating is already sufficient to create alignment within the network (Figure 16b_2_) when compared to isotropic freezing (Figure 16b_1_), multiple freeze‐thawing cycles are commonly used to achieve upper boundaries as far as alignment.^[^ ^39^, ^40^, ^146^, ^246^, ^247^, ^248^, ^249^ ^]^ For instance, a maximum orientation index of 0.8 can be achieved through ice templating.^[^ ^40^ ^]^

Ice‐templated cellulose‐based hydrogels possess similar yield strength upon compression, regardless the stress direction, but they show a remarkably high anisotropy in term of toughness. Their hierarchical features dissipate energy more efficiently than their continuous counterparts due to a higher tortuosity of the assembled elements that leads to a longer path for fracture propagation and to a better stress dissipation mechanism across the elementary building blocks, which leads to specific stress values much lower than the one applied in the macrostructure. Such hydrogels are stiffer in the parallel direction of the tubular architecture, while tougher in the perpendicular direction (Figure 16c).^[^ ^250^ ^]^ Whereas the extended elastic regime at the parallel direction may enable shape‐recovery features in the hydrogel, the compression and buckling of tubular cellular architectures found in the perpendicular direction intensify the energy dissipation across the hydrogel network until large strains, where no abrupt breakage takes place, only progressive compaction of the network.

Although 3D printing has been used toward more complex and truncated objects,^[^ ^100^, ^185^ ^]^ it also has the capability of building hierarchically, aligned structures resembling wood.^[^ ^251^ ^]^ As discussed earlier, direct ink writing, especially, has shown to induce long‐range alignment of cellulose‐based nanoparticles due to shear stresses.^[^ ^108^ ^]^ Shear‐induced alignment is tethered to the geometry of the shearing element.^[^ ^89^, ^91^, ^252^ ^]^ In most of the 3D printing efforts, however, the hydrogel is an intermediate state for the dried, final material.^[^ ^4^, ^101^ ^]^

Hydrogels have been recently designed to display stimuli‐driven, shape‐morphing response. In such systems, external stimuli such as light, chemical environment or humidity can trigger dynamic changes led by the microstructural anisotropic of the material’s architecture.^[^ ^108^, ^253^ ^]^ Vegetal tissues, including wood, are natural analogues of such systems, where bulk deformations are driven by structural and chemical anisotropies found in their plant tissues and cell walls. Relevant to this review is the fact that wood displays a clear directional dependency as far as swelling over humidity changes (Figure 16d). It is well known that section of wood (tangential, radial and longitudinal) display different swelling capacity due to a specific arrangement of elongated cells within the fibers, and subsequently to the ordering of the fibers within the wood. In practical terms, the tangential plane (plane making the tangent of the wood circular cross section) swells/shrinks almost twice more compared to that of the radial plane (plane parallel to the pith‐bark direction) (Figure 16e). While the longitudinal plane (length of the tree) displays nearly negligible swelling.^[^ ^36^, ^41^ ^]^ Such microstructure‐dependent features in wood have been used to prepare humidity‐responsive actuators with preprogrammed movement influenced by the direction within the tangential–radial dimensional space to which the veneers (thin wood slices) are isolated (Figure 16d).^[^ ^254^ ^]^

Likewise following observations in natural wood “actuators” and other plant tissues, such as pinecones and flowers,^[^ ^255^, ^256^ ^]^ nanocelluloses‐based hydrogels can be designed for shape‐morph response over humidity changes.^[^ ^108^ ^]^ Controlled hygroexpansion (and thermal expansion) could be achieved by using a composite ink comprising CNFs embedded in an acrylamide matrix, mimicking the composition of the plant cell wall. Controlling locally the orientation of the fibrils within the acrylamide matrix it is possible to systematically program the elastic behavior and swelling anisotropies of the printed hydrogel (Figure 16f). Therefore, soft actuators can be obtained by printing such composite inks into truncated or complex 3D objects (Figure 16g).^[^ ^108^ ^]^

Cellulose‐based hydrogel actuators mimicking plant’s tissues could be also prepared to act upon changes in the acidic–basic conditions of the surrounding environment. For example, such efforts have considered bilayers of cellulose/carboxymethylcellulose and chitosan chemically crosslinked within each layer and strongly adhered by ionic interactions between layers. In such case, pH‐dependent water interactions and swelling maxima within each layer occur due to differences in protonation and deprotonation of the layers of negatively charged cellulose and the positively charged chitosan. At acid pH, swelling is favored in the chitosan layer, causing the hydrogel to bend in small angles and compress the cellulose layer. The opposite is found at pH neutral to basic. Playing with the arrangement of the layers, such mechanically robust hydrogels can be made to rapidly shape‐morph into rings, tubules, helices and waves, in a repeatedly and fully reversibly manner.^[^ ^38^ ^]^

## 9 Other Wood‐Based Biopolymers for Hydrogels

While the bulk of the efforts toward wood‐ and plant‐based nanocolloids of multiscaled, hierarchical structures exploits the structural feature of nanocelluloses, there is a growing trend to use other plant biomolecules. Hence, here we succinctly consider non‐cellulosic plant components. Their various benefits may be exploited following the inherent functional design in the plants (fulfilling functions such as UV‐resistance, immune responses, and water retention and permeation). Furthermore, either with biocolloids or biopolymers, specific affinities have the potential to enable new property spaces. Efforts are expanding and are expected to synergize and further stimulate the use of biocolloids in a wider array of applications. We herein introduce some notable examples.

**Figure 17:**
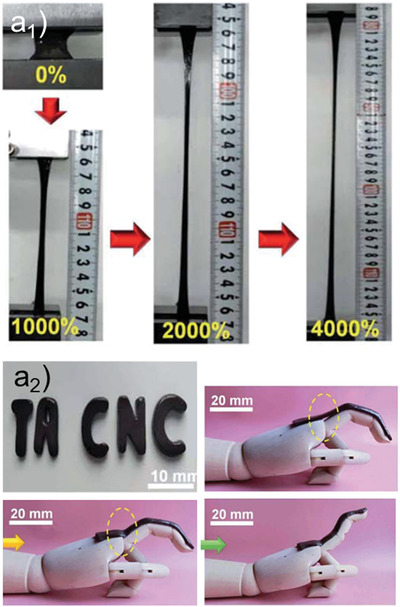
Natural skin‐inspired cellulose‐based biomimetic hydrogel. a_1_) Stretchability of skin inspired TA‐based hydrogels and a_2_) the adaptability of the hydrogel to nonlinear dynamic surfaces. Reproduced with permission.^[^ ^265^ ^]^ Copyright 2019, Royal Society of Chemistry.

We first consider tannin and lignin. Typically, a high density of phenolic hydroxyl groups is present in tannins while lignins have a lower density but a similar UV‐absorption capability. Both possess a great anti‐oxidant activity. In recent efforts, a wide array of lignin^[^ ^257^ ^]^ and tannin‐based^[^ ^258^, ^259^ ^]^ particles have been introduced. Although their potential is not exploited yet in the context of hydrogels, this is to be expected. Tannins and particularly tannic acid (TA) have been widely reported to form biomimetic hydrogel nanocomposites. Hydrogen bonding, electrostatic interaction, π–π, hydrophobic, and coordination are some of the driving forces for tannin self‐assembly.^[^ ^260^, ^261^, ^262^ ^]^ The rapid assembly of TA and group IV metal ions forms metallogels with tunable mechanical properties, optical transparency, and stability in the pH range of 2–10.^[^ ^263^ ^]^ Hydrogels with shape memory behavior and excellent mechanical properties are fabricated through self‐assembly of poly(vinyl alcohol) (PVA) and TA‐coated CNCs at room temperature. The extent of elongation and shape recovery of PVA–TA hydrogels can reach up to 1100%.^[^ ^261^ ^]^ PVA and TA interaction through donation and acceptance of H‐bonding retain the permanent shape, while melting of the PVA crystallites and reversible crystallization contributes to temporary shape.^[^ ^260^, ^261^, ^264^ ^]^ The PVA–TA shape memory hydrogels achieve tensile strength and elastic modulus as high as 84 and 30 kPa with 1 wt% PVA–TA concentration in water.^[^ ^260^ ^]^ Furthermore, a biomimetic skin‐inspired nanocomposite hydrogel was developed through formation of nanohybrids by crosslinking PVA to CNC coated with TA, followed by anchoring Ag nanoparticles on the surface of the modified CNC. The ultrastretchable biomimetic skin‐like nanocomposite recovers 98.6% of the 4000% stretched structure within 10 min. It had a high adhesiveness as well as mechanically and electrically induced self‐healing properties (**Figure** **17**a_1_). The reconfiguration ability to adapt to nonlinear and dynamic surfaces for self‐healing hydrogel sensor application is demonstrated in Figure 17a_2_. The presence of Ag nanoparticles and gallol groups present in TA develop the antimicrobial behavior as well as repeatable adhesiveness to various substrates.^[^ ^265^ ^]^ The water‐resistance adhesion of plant derived tannins has been an inspiration to develop bioadhesives through Michael addition of TA and gelatin under oxidizing condition and silver nitrate crosslinking. Both TA and Ag nanoparticles (reduced from silver nitrate) ensure antimicrobial effects in the hydrogel network. Also, the oxidized polyphenols in TA facilitate wet tissue adhesion through catecholamine‐like chemistry. The tannin‐mimic gelatin hydrogels address the issue related to neurological effects caused by mussel‐inspired dopamine adhesives.^[^ ^266^, ^267^ ^]^ Following similar inspiration of mussel adhesives, TA performs as a crosslinker to develop adhesive gelatin hydrogels with self‐healing properties under alkaline conditions. In comparison to previous work where Ag, temperature, or pH were used altered the gelation time,^[^ ^268^ ^]^ NaIO_4_ oxidant has been used to adjust the gelling time and overall mechanical performance.^[^ ^269^ ^]^

To a less extent compared to tannins, multiple functional phenolic and carboxylate groups exist in lignin, which can perform as radical scavengers and reducing agents.^[^ ^270^ ^]^ Although one of their main uses, it is noteworthy that most adhesives based on lignins and their derivatives are nonrebondable and short term.^[^ ^271^ ^]^ Recently, a plant inspired catechol‐chemistry‐based hydrogel was developed with high toughness, long lasting adhesion, and antibacterial property. Silver ions and lignin nanoparticles trigger a dynamic redox catechol chemistry and form a dynamic redox system that creates long term reductive oxidative environment inside the hydrogel network. In addition, generation of free radicals activates self‐gelation of the hydrogel under ambient condition.^[^ ^270^ ^]^ The presence of lignin is proven to be essential in wood biomimetic hydrogel beads to achieve high pH and thermal stability in enzyme immobilization.^[^ ^272^ ^]^ Hydrogels formed from lignin particles were showed to enhance catalytic efficiency for multiphase reactions as induced by the nanoconfinement as present in the packed particles.^[^ ^273^ ^]^ These recent outcomes infer that controllable properties of lignin hydrogel can be applied in biocatalytic and bioelectronic fields.

Lignin has shown to reduce the incidence of colon cancer. As a natural antioxidant, lignin absorb carcinogens and eliminate the probability of developing cancerous polyps by reducing the resident and exposure time in colon.^[^ ^3^, ^274^ ^]^ Several factors including the source of lignin, pH, temperature and the nature of the solvent has been investigated to predict the assembly of isolated lignin into biomimetic structures, however, more studies are required since several details are not yet fully understood.^[^ ^275^, ^276^, ^277^ ^]^

Biomimetic inks based on hemicelluloses were developed. In plant cell microenvironments, the intrinsic affinity of cellulose to heteropolysaccharides results in a structure that benefits from both flexibility and strength. Similarly, the affinity between these biopolymers can be used to modify the rheological behavior of blends, for example, with TEMPO‐oxidized CNFs and galactoglucomannan methacrylates. Indeed, they have been used to develop UV curable, 3D printable formulations.^[^ ^278^ ^]^ Previously, similar inspiration resulted in the development of biomimetic hemicellulose‐reinforced hydrogels with addition of nanocellulose or through extension of molecular chains for tissue engineering and wound dressing applications. The mechanical properties of the hydrogel was adjustable by the charge density of the nanocellulose, altering the type of hemicellulose, or the chain extension.^[^ ^279^, ^280^, ^281^, ^282^ ^]^ The existence of free hydroxyl groups in the structure of hemicelluloses eases their derivatization through thiol functionalization, tyramine modification or methacrylate derivatization, offering different crosslinking strategies.^[^ ^283^ ^]^

With a rising interest, its high availability, low cost, easy functionalization, and the low immunogenicity of pectin, this biomolecule is a promising candidate in hydrogels for cancer treatment, cartilage and bone tissue regeneration, extracellular matrix mimetic, gene therapy, and drug delivery.^[^ ^284^, ^285^, ^286^ ^]^ In such biomimetic models, pectin affects the architecture and mechanical properties of the hydrogels. Inspired by plant cells, BNC was synthesized in the presence of pectin solutions. Pectin was demonstrated to impact compression and oscillatory deformations despite the lack of tight interactions with BNC. Major changes were observed in the properties before and after hydrogel washing.^[^ ^287^ ^]^ Cartilage tissue engineering benefitted from biomimetic in situ injectable hydrogels based on HA/pectin. The fast gelation rate (e.g., higher rate than gelatin), controllable mechanical properties, adjustable degradation behavior, and tailorable compatibility with tissue provided a suitable tissue mimetic microenvironment to host and maintain chondrocyte phenotype and to enhance the chondrogenesis in encapsulated chondrocytes.^[^ ^288^, ^289^, ^290^ ^]^

## 10 Outlook

While many reviews are available in the area of “hydrogels,” much less information exists in relation to those derived from plant components and especially in connection with the subjects considered in this review. For example, nanocelluloses form hydrogels that are strikingly simple to produce and process. Yet, they can be highly sophisticated, mainly because the possibility of exploiting the multiscale features of related biocolloids, which decode the properties present in the original, natural assemblies. Materials that can be developed from plant‐based hydrogels include foams, sponges, cryogels, xerogels and aerogels. They can be engineered by exploiting their strong interactions with water and for this reason we embarked in an in‐depth discussion of the main associated features, highlighting the far‐reaching impacts of hydrogels. To end, we wish to indicate our focus on phenomenological aspects that factored the role of water, intimately linked to hierarchy in plants. The potential of using such abundant resource and inherent interactions for the development of advanced materials, while reaching performance metrics and cost, are very promising aspects. We provided a flavor for promising solutions that factor (plant‐based) material performance. Related aspects are most relevant, in the simplest form, in the next generation superinsulating materials that can be developed by drying the given hydrogel precursor, which is expected to be bio‐based and obtained by green routes. In the other extreme, as far as more complex and elegant applications, the multiscale interaction of the source materials can be suited in complex designs, to some extent mimicking the functions of plants. An example is fluid transport for filtration, desalination, and heat/sound and light management. One exquisite descriptive example is that of opto‐mechanical responsive materials formed from hydrogels that preserve the chiral nematic arrangement of CNC, as presented in previous sections. Such architectures are uniquely formed by biocolloids, and exemplify future directions in the utilization of new classes of materials and even metamaterials. Particularly, plant‐based nanocellulose as well as BNC is already being reported for their possibilities in opening new property spaces. A present challenge is the fact that these nanomaterials are not widely available at an industrial scale. While many units at the pilot, demonstration and larger scales are being reported, the development of hydrogels derived from plant components needs time to meet a high commercial relevance. Some commercial examples of nanocellulose‐based hydrogels currently exist and major advances in the direction of commercialization are expected in the future. Thus, plant‐based hydrogels are not yet used in our daily life, although they possess outstanding structural properties. There are many examples of cellulose‐based hydrogels (sponges, acetates and derivatives), but the bigger share of biocolloids is yet under development. As is the case of other developments, it is a question of time for plant‐based hydrogels to enter as substitutes of already existing materials as well as options to develop completely new ones.

As part of our food diet, polysaccharides are “human‐friendly” polymers and present themselves with plenty of opportunities for application in life sciences. However, their accessibility and low cost also means that they are suitable for the next generation, common use bio‐based plastics, wearables and structural materials. Here, the adoption of plant‐based structures offers a unique opportunity in the design of new “smart” materials, similar or better than “classical” or synthetic ones. Taking advantage of the structure of a hydrogel, outstanding structural properties can be developed from the very fine porosity, from wet to solid networks, with pores that can be tailored in size and distribution. A question that is always present is the control of property profiles. Such aspect is integral to any utilization of natural materials. Therefore, it is expected that research interest will develop around mechanisms to achieve uniform or controllable morphology, size and chemical composition. For this, new fractionation approaches are probably needed for processing plant components. As discussed herein, water interactions with plant materials can be quite diverse and complex. In fact, this is a subject that is at the center of active research that use computational tools combined with new techniques to access the ultrastructure of relevant cellulosic structures. Achieving control on water interactions is becoming increasingly critical for the design and use of hydrogels. In closing, plant‐based hydrogels are very versatile for their assembly and morphology combined to the possibility of functionalization. Future developments in this area may be quite exciting and surprising.

## Conflict of Interest

The authors declare no conflict of interest.
